# Synthesis, Characterization, Computational Evaluation, CO-Releasing Properties, and Molecular Docking Interactions of New [Mn(CO)_3_(bpy)L]PF_6_‑Type Molecules

**DOI:** 10.1021/acsomega.5c03085

**Published:** 2025-07-14

**Authors:** Sena Ceren Önbaş, Goncagül Serdaroğlu, Neslihan Şahin, Elvan Üstün, İsmail Özdemir

**Affiliations:** † Department of Chemistry, Faculty of Art and Science, Ordu University, Ordu 52200, Turkey; ‡ Math. and Sci. Edu., Faculty of Education, Cumhuriyet University, Sivas 58140, Turkey; § Department of Chemistry, Faculty of Science and Art, İnönü University, Malatya 44280, Turkey

## Abstract

Designing CO-releasing molecules, which store, transport, and release carbon monoxide in the target tissue, has accelerated since scientists revealed that carbon monoxide is one of the transmitters and could be effective in treatment procedures. The most important candidates for this task are metal carbonyl complexes. In this study, [Mn­(CO)_3_(bpy)­L]­PF_6_-type metal carbonyl complexes were synthesized and characterized and the CO-releasing activities of these molecules were investigated. In addition, the optimization and theoretical analysis of the molecules were performed with DFT/TDDFT-based calculation methods. DFT computations at the B3LYP/6–311G­(d,p)/LANL2DZ level were performed to assign vibrational modes and NMR shifts following geometry optimization and confirmation. Moreover, NBO analyses were performed to predict the important electronic interaction that occurred in complexes: the results implied that the biggest contribution could come from the resonance interactions. FMOs analyses indicated that the 2e could be a softer (η= 1.511 eV) and is less stabilized complex via back-donation (Δε_back‑donat._ = −0.378 eV). Additionally, the interactions of the molecules with HSA, BSA, and DNA were investigated with molecular docking methods, and the binding properties of the manganese complexes were analyzed in vitro with UV–vis spectroscopy against BSA and DNA by the Benesi–Hildebrand method.

## Graphical abstract

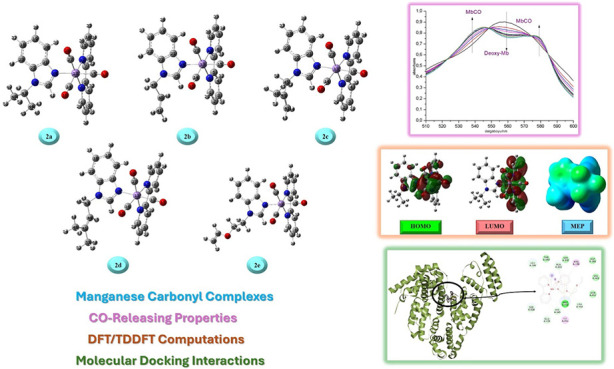

## 1 Introduction

Carbon monoxide (CO), which occurs as a byproduct in the oxidative metabolism of hemoglobin, is well-known to the human body. After the discovery of the healing effects of nitrogen monoxide (NO) as a transmitter, CO attracted the attention of the scientific world,^1,2^ and the possible effects of CO started to be analyzed in the early 2000s.^3,4^ CO has been used in vasodilatory and organ transplantation treatment procedures.^5,6^ In addition, several properties of CO such as anticancer, antiapoptotic, antioxidant, and antimalarial properties have been investigated, and promising results have been obtained.^7,8^ The main obstacle to the use of CO as a treatment agent, even in hospital environments, is the difficulty in controlling the level of CO supplementation. Molecules, which store CO, transport it to the target tissue, and release it in a regular, controllable, and predictable manner, are called CORMs (CO-releasing molecules), and metal carbonyl complexes are among the best candidates for this task.^9^ Several transition metals such as Mn, Re, Ru, and Fe have been more frequently analyzed for the CO releasing properties of their carbonyl complexes since a CO-releasing molecule must dissolve in a biocompatible solvent, adapt to analysis conditions, and be easily modified with different substituents. Manganese complexes are advantageous due to both their ease of binding and ionic properties, and manganese-based CORMs have been studied by different research groups.^10–12^ On the other hand, the characteristics of the secondary ligands could change the CO-releasing activities of metal carbonyl complexes. Benzimidazoles, one of the important groups of the azoles family, have been frequently studied for their antibacterial, antiviral, and anticancer properties.^13^ Therefore, in this study, [Mn­(CO)_3_(bpy)­L]­PF_6_-type metal carbonyl complexes (bpy: 2,2’-bipyridyl; L: 1-isopropylbenzimidazole, 1-allylbenzimidazole, 1-(2-metallyl)­benzimidazole, 1-(3,3-dimethylallyl)­benzimidazole, 1-(2-vinyloxyethyl)­benzimidazole) were analyzed.

As is known, DFT methods are widely used to predict a broad range of properties such as structural, electronic, and magnetic properties of metal complexes and provide very useful information in terms of predicting processes that are quite costly and time-consuming to determine experimentally.^14,15^ In this respect, Boro and coworkers have reported a series of Mn­(II) and Cu­(II) complexes decorated with pyrazole-based ligands and evaluated the energetic characteristics of π···π stacking, H-bonding, and noncovalent interactions in light of DFT computations.^16^ Also, Wang and coworkers have performed the ωB97X-D/6–311G**/LANL2DZ computations on NN-Mn-CO complexes to evaluate the catalytic activity mechanism.^17^ Also, the possible interaction energies of triazine-modified Mn­(II) complexes with 1,3,5-triazine have been calculated with the DFT/ωB97XD/6–311G­(d,p)/LANL2DZ level of theory.^18^ Recently, Wani and coworkers have investigated using the PBE0-D3/def2-TZVP level the reaction mechanism of the hydrophosphination of olefins catalyzed with MnBr­(CO)_5_.^19^ Avcı and coworkers have performed CAM-B3LYP and ωB97XD level computations at the 6-311+G­(d,p)//LanL2DZ basis set to predict the optical and nonlinear optical properties of the newly synthesized azide-based metal complexes including Mn­(II) complexes.^20^ In this work, DFT/B3LYP/6–311G** level simulations of **2a**–**e** were performed to evaluate chemical reactivity features following structural confirmation, NMR shifts, and FT-IR characterizations. Molecular docking, which is considered an essential technique in drug analysis/discovery, allows us to obtain important results on the interaction of both main products and intermediates with the tissues by using target crystal structures.^21,22^ In this study, the interactions of molecules with HSA, BSA, and DNA were investigated by molecular docking methods. Albumin is one of the most abundant proteins found in humans and other mammals. It plays a crucial role in the transport of a wide range of endogenous and exogenous substances and is essential for maintaining the colloid osmotic pressure of the blood. Additionally, albumin is significant in pharmacokinetic and toxicokinetic processes related to enzymatic activity.^23^ DNA is fundamental to biological functions as it contains the genetic information required for the synthesis of all proteins and enzymes. Since the discovery of its structure, DNA has become a key target for many therapeutically relevant small molecules including anticancer agents and antibiotics. These small molecules can bind directly to the DNA strand or interact with proteins associated with DNA.^24^ Therefore, DNA- and BSA-binding characteristics could provide useful foresight about the drug candidacy of a molecule. Additionally, the binding properties of the manganese complexes were analyzed in vitro with UV–vis spectroscopy against BSA and DNA.

## 2 Experimental and Computational Methods

### 2.1 Material and Measurement

All the synthesis processes were performed with Schlenk techniques under an argon atmosphere. All spectrophotometric measurements were conducted in the dark due to the sensitivity of the molecules. The FIR spectra were obtained using a Shimadzu IRAffinity-1 instrument, while all UV–vis spectra were recorded with a Shimadzu UV-1800. ^1^H NMR and ^13^C NMR spectra were recorded using Bruker Avance AMX and Bruker Avance III spectrometers operating at 400 MHz (^1^H NMR) and at 100 MHz (^13^C NMR) in CDCl_3_ with TMS added. NMR multiplicities are abbreviated as follows: s = singlet, d = doublet, *t* = triplet, and *m* = multiplet signal. The chemical shifts (δ) are reported in ppm. Coupling constants (*J* values) are given in hertz (Hz).

### 2.2 Synthesis and Characterizations

#### 2.2.1 Synthesis of the Complexes

The synthesis procedure and the characterization details of the ligands are presented in the Supporting Information.^25–28^ In diethyl ether, Mn­(CO)_5_Br (1 mmol) and an excess amount of 2,2’-bipyridyl (1.4 mmol) were refluxed for 2 h, and Mn­(CO)_3_(bpy)Br crude product was filtered with a por4 filter paper and dried under vacuum.^29^ AgOTf (1.4 mmol) was added to the solution of Mn­(CO)_3_(bpy)Br (1.0 mmol) in acetone (15 mL), and AgBr, which precipitated after 1 day of stirring at room temperature, was filtered with Celite. The benzimidazole-type ligand (1.2 mmol) was added to the clear solution, and the final mixture was stirred until the product was clearly recorded in FT-IR spectra. After the crude product emerged, acetone was evaporated. For achieving the final product with a higher yield, the OTf anion was replaced with a PF_6_ anion in methanol, and the final product was filtered and washed with cold diethyl ether; the final complex was dried under vacuum nearly for 4 h and stored in an amber glass container in the dark under argon (Scheme 1).

**Scheme 1:**
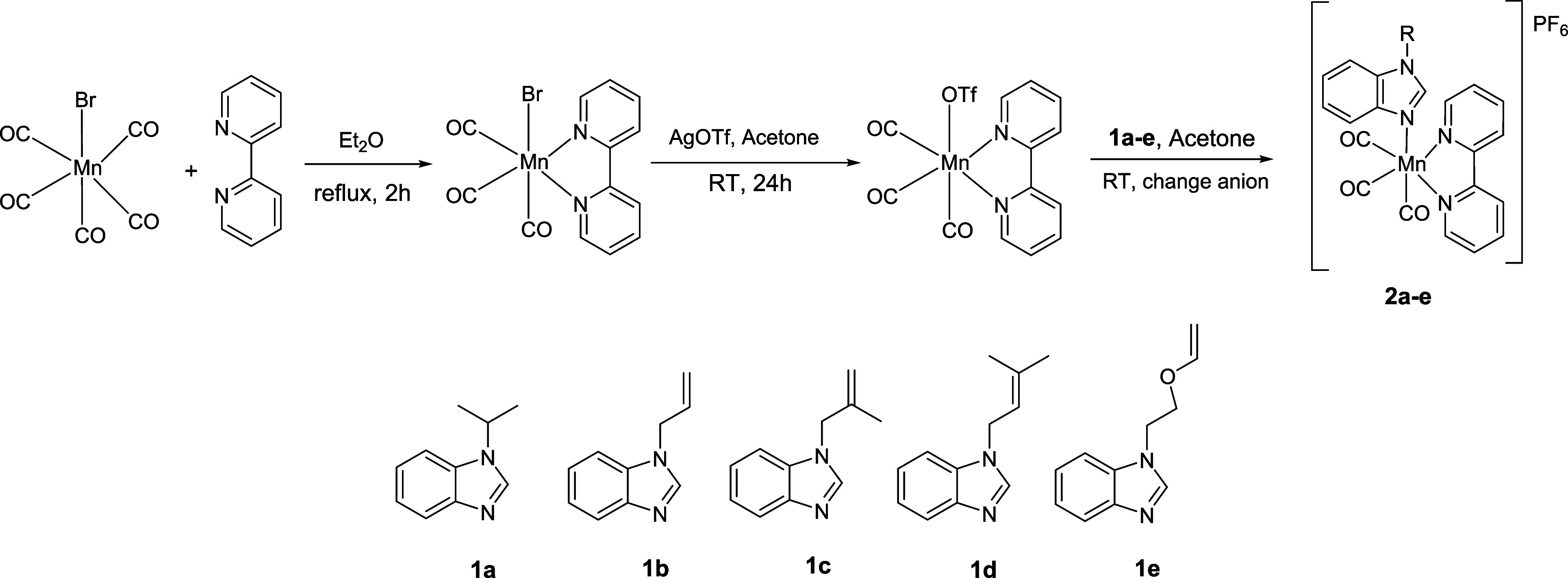
Reaction Pathway of **2a**–**e**

##### 2.2.1.1 [Mn­(CO)_3_(bpy)­(1-isopropylbenzimidazole)]­PF_6_, [**2a**]

Yellow solid. Yield: 404 mg (67%). Elemental analysis, calcd for C_23_H_20_F_6_MnN_4_O_3_P.2H_2_O: C: 43.55, H: 4.35, N: 8.24 (%); found: C: 43.21, H: 4.26, N: 8.76 (%). ^1^H NMR (400 MHz, DMSO) δ 9.48 (d, *J* = 5.1 Hz, 2H, NC_10_ *H*_8_N), 8.63 (d, *J* = 8.0 Hz, 2H, NC_10_ *H*_8_N), 8.31 (t, *J* = 7.5 Hz, 2H, NC_10_ *H*_8_N), 7.90–7.80 (m, 2H, NC_10_ *H*_8_N), 7.67 (s, 1H, NC*H*N), 7.55 (d, *J* = 8.7 Hz, 1H, NC_6_ *H*_*4*_N), 7.35 (d, *J* = 16.4 Hz, 1H, NC_6_ *H*_*4*_N), 5.12 (t, *J* = 6.5 Hz, 1H, NC_6_ *H*_*4*_N), 4.95 (d, *J* = 6.4 Hz, 1H, NCH­(C*H*_3_)_2_) 1.61 ve 1.55 (s, 6H, NCH­(C*H*_3_)_2_). ^13^C NMR (101 MHz, DMSO) δ 155.53, 155.22, 145.58, 141.98, 140.91 (N*C*_8_H_10_N), 139.11, 137.93, 133.72, 128.32, 124.55, 123.93 (NC_6_ *H*_*4*_N), 122.91 (N*C*HN), 43.27 (N*C*H­(CH_3_)_2_) 25.72, 18.06 (NCH­(*C*H_3_)_2_). LCMS: *m*/*z* 439.01 [M–CH_3_–PF_6_]^+^. IR (cm^–1^, ATR): 1605 (m, C–N), 1929, 2029 (s, CO).

##### 2.2.1.2 [Mn­(CO)_3_(bpy)­(1-allylbenzimidazole)]­PF_6_, [**2b**]

Yellow solid. Yield: 430 mg (72%). Elemental analysis, calculated for C_23_H_18_F_6_MnN_4_O_3_P: C: 46.68, H: 3.23, N: 9.90 (%); found: C: 46.17, H: 3.03, N: 9.36 (%). ^1^H NMR (400 MHz, DMSO) δ 9.49 (s, 2H, NC_10_ *H*_8_N), 8.62 (d, *J* = 6.8 Hz, 2H, NC_10_ *H*_8_N), 8.30 (s, 2H, NC_10_ *H*_8_N), 7.83 (s, 2H, NC_10_ *H*_8_N), 7.60 (s, NC*H*N), 7.36 (s, 2H, NC_6_ *H*_*4*_N), 5.77 (s, 2H, NC_6_ *H*_*4*_N), 5.11 (t, *J* = 43.3 Hz, 4H, NC*H*_2_CHC*H*_2_), 4.79 (s, 1H, NC*H*_2_CHC*H*_2_). ^13^C NMR (101 MHz, DMSO) δ 155.27, 146.50, 141.80, 140.90, 133.85 (NC_10_ *H*_8_N ve NC_6_ *H*_*4*_N), 132.48 (NCHN), 124.80, 124.29, 117.75, 113.22 (N*C*H_2_CHCH_2_), 47.09 (N*C*H_2_CHCH_2_). LCMS: *m*/*z* 452.1 [M-PF_6_]^+^. IR (cm^–1^, ATR): 1605 (m, C–N), 1936.5, 2037 (s, CO)

##### 2.2.1.3 [Mn­(CO)_3_(bpy)­(1-(2-metallyl)­benzimidazole)]­PF_6_, [**2c**]

Yellow solid. Yield: 388 mg (63%). Elemental analysis, calculated for C_24_H_20_F_6_MnN_4_O_3_P: C: 46.57, H: 3.57, N: 9.67 (%); found: C: 46.84, H: 3.57, N: 9.10 (%). ^1^H NMR (400 MHz, DMSO) δ 9.54–9.43 (m, 2H, NC_10_ *H*_8_N), 8.60 (d, *J* = 8.2 Hz, 2H, NC_10_ *H*_8_N), 8.28 (t, *J* = 7.6 Hz, 2H, NC_10_ *H*_8_N), 7.86–7.78 (m, 3H, NC_10_ *H*_8_N ve NC*H*N), 7.33 (d, *J* = 5.7 Hz, 4H, NC_6_ *H*_*4*_N), 4.96 (s, 1H, NCH_2_C­(CH_3_)­C*H*_2_), 4.74 (s, 1H, NCH_2_C­(CH_3_)­C*H*_2_), 4.37 (s, 2H, NC*H*_2_C­(CH_3_)­CH_2_), 1.66 (s, 2H, NCH_2_C­(C*H*_3_)­CH_2_). ^13^C NMR (101 MHz, DMSO) δ 155.61, 146.81, 141.20 (NC_10_ *H*_8_N), 133.18 (N*C*HN), 128.31, 124.91, 117.80, 112.91 (NC_6_ *H*_*4*_N), 50.54 ((N*C*H_2_C­(CH_3_)­CH_2_)), 19.77 ve 19.08 (NCH_2_ *C*(*C*H_3_)­CH_2_). LCMS: *m*/*z* 473.1 [M–PF_6_]^+^. IR (cm^–1^, ATR): 1605 (m, C–N), 1929, 2037 (s, CO)

##### 2.2.1.4 [Mn­(CO)_3_(bpy)­(1-(3,3-dimethylallyl)­benzimidazole)]­PF_6_, [**2d**]

Yellow solid. Yield: 408 mg (65%). Elemental analysis, calculated for C_25_H_22_F_6_MnN_4_O_3_P.H_2_O: C: 47.02, H: 4.31, N: 8.99 (%); found: C: 47.11, H: 3.95, N: 8.79 (%). ^1^H NMR (400 MHz, DMSO) δ 9.58 (d, *J* = 5.2 Hz, 2H, NC_10_ *H*_8_N), 8.52 (d, *J* = 8.0 Hz, 2H, NC_10_ *H*_8_N), 8.26 (t, *J* = 7.5 Hz, 2H, NC_10_ *H*_8_N), 7.76 (dd, *J* = 46.2, 15.3 Hz, 2H, NC_6_H_4_N), 7.31 (d, *J* = 7.8 Hz, 3H, NC*H*N ve NC_6_H_4_N), 1.57 (s, 3H, NCH_2_CHC­(C*H*_3_)_2_), 1.29 (s, 3H, NCH_2_CHC­(C*H*_3_)_2_). ^13^C NMR (101 MHz, DMSO) δ 155.50, 144.06, 141.53, 140.71, 132.97, 128.22, 124.34 (N*C*_10_H_8_N), 123.21 (N*C*HN), 122.56, 119.84, 117.58, 113.11, 111.63 (NC_6_H_4_N), 48.8­(N*C*H_2_CHC­(CH_3_)_2_), 22.60, 21.97 NCH_2_CHC­(*C*H_3_)_2_. LCMS: *m*/*z* 469.1 [M–CH_3_–PF_6_]^+^. IR (cm^–1^, ATR): 1605 (m, C–N), 1936.5, 2037 (s, CO)

##### 2.2.1.5 [Mn­(CO)_3_(bpy)­(1-(2-vinyloxyethyl)­benzimidazole)]­PF_6_, [**2e**]

Yellow solid. Yield: 442 mg (70%). Elemental analysis, calculated for C_24_H_20_F_6_MnN_4_O_4_P·0.3Et_2_O: C: 46.55, H: 3.92, N: 9.10 (%); found: C: 46.31, H: 4.01, N: 8.57 (%). ^1^H NMR (400 MHz, DMSO) δ 9.41 (d, *J* = 5.2 Hz, 2H, NC_10_ *H*_8_N), 8.58 (d, *J* = 7.9 Hz, 2H, NC_10_ *H*_8_N), 8.26 (t, *J* = 7.4 Hz, 2H, NC_10_ *H*_8_N), 7.61 (s, 1H, NC*H*N), 7.32 (d, *J* = 9.1 Hz, 2H, NC_10_ *H*_8_N), 4.63 (s, 1H, Ar-*H*), 4.42 (s, 2H, Ar–*H*), 4.21 (d, *J* = 14.2 Hz, 2H), Ar-*H*, 4.07 (s, 1H, Ar-*H*), 3.99 (d, *J* = 9.5 Hz, 2H, NCH_2_C*H*_2_O), 3.86 (d, *J* = 6.6 Hz, 1H, OCHC*H*_2_), 3.78 (s, 2H, NCH_2_C*H*_2_O). ^13^C NMR (101 MHz, DMSO) δ 155.10, 154.79, 151.25, 150.88, 146.24 (NC_10_H_8_N), 141.17 (NCHN), 140.57, 133.31, 127.91, 123.94, 123.84, 117.18, 112.48 (NC_6_H_4_N), 87.72 (N*C*H_2_CH_2_OCHCH_2_), 66.10 (N*C*H_2_CH_2_OCHCH_2_), 65.21 (N*C*H_2_CH_2_OCHCH_2_), 44.25 (N*C*H_2_CH_2_OCHCH_2_). LCMS: *m*/*z* 485.1 [M–PF_6_]^+^. IR (cm^–1^, ATR): 1620 (m, C–N), 1929, 2029 (s, CO)

#### 2.2.2 Myoglobin Assay

The myoglobin solution, which was prepared in PBS (pH 7.4, 0.1 M), was reduced to deoxymyoglobin using sodium dithionite (100 mM). The solutions of each complex (15 mM) in DMSO were incubated with deoxymyoglobin solution (60 mM), and the final solution was exposed to 365 nm UV light from a distance of 2 cm. Deoxymyoglobin has a band at 557 nm, while carbonmonoxy-myoglobin has two identical bands at 540 and 577 nm, and the changes at these wavelengths make the process observable and determinable. Thus, the CO-releasing properties of the molecules were calculated using the Lambert–Beer Law.

### 2.3 Computational Methods

All DFT computations of **2a**–**2e** were conducted at the B3LYP^30,31^ level and the 6–311G­(d,p)^32,33^ basis set for C, O, N, and H atoms, and LANL2DZ^34^ for Mn­(I) by the G09W^35^ package. The simulated vibrations of the complexes were scaled by 0.9619 ^36^ to make them comparable with the recorded modes. Also, NMR shifts based on the GIAO “Gauge-Independent Atomic Orbital” ^37,38^ method were calculated in DMSO as the solvent, which was the same as the experimental environment, using the C-PCM “Conductor-like Polarizable Continuum Model”.^39^ The optimized structures, FMO amplitudes, and MEP plots of complexes **2a**–**e** were visualized using the GaussView 6.0 package.^40^

The NBO^41^ analysis encoded in G16W was performed to predict the intramolecular interactions that contributed to the decreasing stabilization energy (*E* ^(*2)*^).^42–44^ Also, global reactivity scores of **2a**–**e** were computed using the B3LYP and PBEPBE^45^ levels of DFT. The *I* → “ionization energy” and *A* → “electron affinity” depending on Koopmans theorem^46^ are used to predict the global reactivity parameters, which are χ → “electronic chemical potential”, η → “global hardness”, ω → “electrophilicity”, Δ*N*_max_ → “the maximum charge transfer capability index”,^47–50^ ω- → “the electrodonating power” and ω+ → “the electroaccepting power”,^51^ and Δ*E*_back‑donat._ → “back-donation energy”.^52^

All optimized structures were confirmed by having no imaginary frequencies at the B3LYP and PBEPBE levels. Also, all DFT computations were performed with the case of the charge and multiplicity of the complexes set to +1 and +1, respectively, due to the charge of the Mn atom being +1.

### 2.4 Molecular Docking Method

AutoDock 4.2 (MGL Tools 1.5.6).^53^ was used for molecular docking performances to determine the interactions of manganese carbonyl complexes with the DNA dodecamer (PDB ID: 1bna), human serum albumin (PDB ID: 1bm0), and bovine serum albumin (PDB ID: 4f5s) crystal structures, which were downloaded from https://www.rcsb.org/.^54–56^ After all the heteroatoms, water molecules, and cofactors were excluded, all the molecules were recorded in PDBQT format.^57^ The target molecule was considered as a rigid molecule with only polar hydrogens. All the docking poses and simulations were visualized using Discovery Studio 4.1.0.

### 2.5 DNA Binding Analysis

DNA-binding properties of the molecules were analyzed with the Benesi–Hildebrand method with UV–vis spectroscopy. The stock solution of manganese complexes (3 mM) was prepared with DMSO, while a fresh stock solution of DNA from commercial CT-DNA was prepared in Tris-HCl buffer solution (pH = 7.4), and the ratio of *A*_260_/*A*_280_ of the DNA stock was recorded as 1.93. A certain concentration of DNA (25 μM) solution was incubated with different amounts of each complex (0, 5, 10, 15, 20, 25, 30, and 35 μM) for 1 h at room temperature. The absorption spectra of all solutions were recorded using a Shimadzu UV-1800 spectrophotometer between 240 and 500 nm.

### 2.6 BSA Binding Analysis

BSA binding analysis of the molecules was performed with UV–vis spectroscopy, and the records were evaluated using the Benesi-Hildebrand equation. The BSA stock solution was freshly prepared in phosphate-buffered saline (pH 7.4), while the stock solutions of the molecules were prepared in DMSO. A certain BSA solution (15 μM) was incubated with each molecule at different concentrations (0, 2, 4, 6, 8, 10, 12, and 14 μM) for 20 min at room temperature. The absorption spectra of all solutions were recorded between 250 and 350 nm.

## 3 Result and Discussion

### 3.1 Synthesis and Characterization

All the molecules were synthesized under an argon atmosphere at room temperature with Schlenk techniques. The molecules were characterized by FT-IR, ^1^H and ^13^C NMR, LC-MS, and elemental analysis, and the outputs of these methods are presented in the Supporting Information.

The LC-MS spectra (Figures S1–S5) and the elemental analysis results (presented in the Supporting Information) of all the complexes were consistent with expectations.

FT-IR spectroscopic methods are among the key tools to predict structural characterization via determining the functional group vibrations in molecular systems. In this work, Table 1 displays the selected calculated functional group vibrations of the studied complexes **2a**–**e** and the recorded spectra of the dataset are given in Figures S1–S5. In the literature, the carbonyl group vibration for the Mn–CO moiety has been specifically recorded within ∼2100–1900 cm^–1^.^58,59^ Although the IR spectra of the complexes should exhibit three CO bands due to the C_S_ point group, the third band of the complexes could be obscured by the second broad and strong carbonyl band. The bands at 1605 and 1620 cm^–1^ could be attributed to the C–N residues of benzimidazoles. The strong bands at 825 cm^–1^ were assigned to P–F stretching frequencies, which could confirm ionic changes as PF_6_ from OTf, which was identified with the bands at 1261, 1229, 1146, and 1030 cm^–1^. The FT-IR spectra of the complexes were also confirmed with the DFT calculation methods. Herein, the νC≡O vibration for **2a**–**e** complexes appeared strongly in the region of 2037–1929 cm^–1^ and was assigned in the range of 2053–1984 cm^–1^. Moreover, the νNC vibration related to the imidazole part of the complexes was recorded in 1620–1605 cm^–1^ and was computed at 1554 cm^–1^ for all complexes. On the other hand, the νNC vibrations for complexes contributed to the bending and νCC stretching modes of the imidazole ring and were predicted in the range of 1554–1033 cm^–1^ with varied intensities. Also, the assigned mode at 1127 and 1070 cm^–1^ for complex **2e** was assigned as the single bond νC–O vibration and was associated with the 2-vinyloxyethyl substitution on the benzimidazole unit. Recently, the νC–O stretching mode for a series of pyrroles was recorded within 1274–1041 cm^–1^ and assigned within 1243–918 cm^–1^ by B3LYP/6–311G** level.^60^ From Table 1, the νC=C stretching for the aliphatic group of complexes **2b**–**e** was determined in the range of 1657–1644 cm^–1^. The νCH_(aromatic)_ vibrations related to the aromatic rings for **2a**–**e** were predicted by DFT in the range of 3144–3062 cm^–1^, whereas the aliphatic C–H modes were determined in the range of 2944–3024 cm^–1^. Furthermore, νCH_2_ and symmetric νCH_3_ stretching modes were estimated in the ranges of 3133–2903 cm^–1^ and 2924– 2908 cm^–1^, respectively. The symmetric elongation modes associated with methyl group(s) (sbCH_3_) for complexes **2a**, **2c**, and **2d** were assigned in the ranges of 1383–1357 cm^–1^, 1369–1365 cm^–1^, and 1370–1361 cm^–1^, respectively. Recently, the sbCH_3_ elongation mode for pyrrole derivatives appeared at 1379 cm^–1^ and was assigned in the range of 1374–1344 cm^–1^.^61^ In previous work, Goswami and coworkers reported the *N*=C stretching mode at 1599 cm^–1^,^62,63^ which is related to the imine group of 1-((((5- bromothiophen-2-yl)­methylene)­hydrazono)­methyl)­naphthalen-2-ol. Also, the recorded peaks at 1575, 1466, and 1416 cm^–1^ were associated with the aromatic νCC stretching mode of the naphthalene and thiophene rings.^62^

**Table 1:** Calculated Frequencies (in cm^–1^), at B3LYP/6-311G­(d,p)/LANL2DZ

	**2a**	**2b**	**2c**	**2d**	**2e**
νCH(_ar._)	3153–3061	3147–3061	3144–3061	3151–3060	3135–3062
νCH_2_	3000–2985	3101–2930	3096–2937	3006–2930	3133–2903
νCH(_al._)	2944	3024	—	3013	3003
νCH_3_	2924, 2921	—	2908	2913,2904	—
νC≡O	2052	2052	2052	2051	2053
νC≡O	1998	1998	1997	1997	1999
νC≡O	1985	1986	1985	1984	1986
νC=C (al.)	—	1644	1649	1657	1644
νCC(ar.)	1591–1235	1592–1235	1592–1235	1592–1235	1593–1235
νNC	1554–1033	1554–1065	1544–1064	1554–1057	1554–1313
sbCH_3_	1383–1357	—	1369–1365	1370–1361	—
νC–O	—	—	—	—	1127,1070

NMR spectroscopy is used to determine the structure of the molecular systems and provide useful information on the structure, dynamics, and chemical environment of the related system. In this study, both 1H and 13C NMR spectra of the complexes were recorded for confirming the structures of the molecules, and the recorded spectra of the dataset are given in Figures S1–S5. Additionally, the NMR spectra of the molecules were analyzed by DFT computational methods. Recently, C shifts related to the C≡O groups for Mn­(I) complexes have been observed in DMSO at 222.86, 221.59, and 219.10 ppm.^61^ From Table 2, the C shifts associated with the carbonyl groups of complexes **2a** were calculated as 242.5 (C14), 239.9 (C15), and 242.6 (C27) ppm in DMSO. The chemical shifts for the C atoms included in the bpy unit of complex **2a**, which are bonded to electronegative nitrogens, were determined to be in the range of 161.7–164.9 ppm (C1, C5, C7, C11). Also, C21, C22, and C25 shifts for **2a** were calculated as 150.2, 141.5, and 150.2 ppm, respectively, which are due to neighboring nitrogens of the benzimidazole unit. The observed signals in the range of 43.27–18.6 ppm for **2a** were assigned as aliphatic C shifts of the isopropyl group for **2a** in the range of 22.1–55.5 ppm.

**Table 2:** ^*13*^ *C* NMR Shifts

**2a**		**2b**		**2c**		**2d**		**2e**	
1-C	161.7	1-C	161.7	1-C	161.7	1-C	161.6	1-C	161.7
2-C	135.1	2-C	135.1	2-C	135.1	2-C	135.0	2-C	135.2
3-C	147.7	3-C	147.7	3-C	147.7	3-C	147.6	3-C	147.8
4-C	130.2	4-C	130.2	4-C	130.2	4-C	130.2	4-C	130.3
5-C	164.9	5-C	164.9	5-C	164.9	5-C	164.9	5-C	165.0
7-C	164.3	7-C	164.4	7-C	164.3	7-C	164.4	7-C	164.3
8-C	129.8	8-C	129.8	8-C	129.8	8-C	129.8	8-C	129.8
9-C	147.2	9-C	147.2	9-C	147.2	9-C	147.2	9-C	147.2
10-C	133.7	10-C	133.7	10-C	133.7	10-C	133.7	10-C	133.7
11-C	162.5	11-C	162.5	11-C	162.5	11-C	162.6	11-C	162.5
14-C	242.5	14-C	242.3	14-C	242.5	14-C	242.5	14-C	242.2
15-C	239.9	15-C	240.0	15-C	239.9	15-C	239.9	15-C	240.0
18-C	130.5	18-C	130.7	18-C	130.7	18-C	130.3	18-C	131.0
19-C	129.6	19-C	129.7	19-C	129.6	19-C	129.6	19-C	129.8
20-C	124.1	20-C	124.1	20-C	124.1	20-C	123.9	20-C	124.4
21-C	150.2	21-C	150.5	21-C	150.4	21-C	150.9	21-C	150.2
22-C	141.5	22-C	141.8	22-C	142.1	22-C	142.0	22-C	141.5
23-C	117.0	23-C	116.9	23-C	116.7	23-C	117.1	23-C	116.9
25-C	150.2	25-C	152.2	25-C	151.9	25-C	151.7	25-C	153.5
27-C	242.6	27-C	242.6	27-C	242.7	27-C	242.6	27-C	242.6
43-C	55.5	41-C	131.4	41-C	126.7	41-C	158.5	44-C	49.3
44-C	22.1	45-C	53.9	45-C	56.7	43-C	48.6	45-C	94.5
48-C	25.3	46-C	139.3	48-C	22.3	44-C	121.0	46-C	162.4
				49-C	151.0	48-C	19.9	47-C	74.2
						53-C	29.2		

For **2a**, the recorded peaks at 9.48 and 8.63 ppm were calculated for the hydrogens (Hs), close to the nitrogen of the bpy unit and the closest carbonyl groups chelated to the Mn­(I), at 9.70 (H29) and 9.89 (H36) ppm (Table 3). Similar proton (H29, H36) shifts for complexes **2b**–**e** were estimated in the range of 9.63–9.89 ppm and appeared in the range of 9.58–8.52 ppm. Also, the signals appearing in the range of 3.78–4.07 ppm in the ^1^H NMR spectrum of **2e** were associated with the 2-vinyloxyethyl group (H42, H48–52) and calculated in the range of 3.94–4.67 ppm. The chemical shifts belonging to the allyl group for 2b were recorded in the range of 4.79–5.77 ppm and simulated in the range of 4.34–5.99 ppm (H42–44, H47, H48).

**Table 3:** ^*1*^ *H* NMR Shifts

	**1a**		**1b**		**1c**		**1d**		**1e**
29-H	9.70	29-H	9.64	29-H	9.63	29-H	9.63	29-H	9.70
30-H	8.02	30-H	7.99	30-H	7.98	30-H	8.00	30-H	8.04
31-H	8.39	31-H	8.40	31-H	8.39	31-H	8.40	31-H	8.40
32-H	8.42	32-H	8.43	32-H	8.43	32-H	8.42	32-H	8.42
33-H	8.34	33-H	8.35	33-H	8.35	33-H	8.34	33-H	8.33
34-H	8.23	34-H	8.24	34-H	8.23	34-H	8.24	34-H	8.22
35-H	7.91	35-H	7.91	35-H	7.90	35-H	7.92	35-H	7.89
36-H	9.89	36-H	9.89	36-H	9.89	36-H	9.89	36-H	9.86
37-H	7.58	37-H	7.59	37-H	7.60	37-H	7.57	37-H	7.60
38-H	7.55	38-H	7.56	38-H	7.56	38-H	7.54	38-H	7.57
39-H	8.11	39-H	8.12	39-H	8.13	39-H	8.06	39-H	8.15
40-H	7.65	40-H	7.65	40-H	7.66	40-H	7.66	40-H	7.68
41-H	7.67	42-H	4.89	42-H	4.22	42-H	4.48	42-H	3.94
42-H	4.52	43-H	5.78	43-H	5.59	45-H	5.41	43-H	6.57
45-H	1.49	44-H	5.99	44-H	5.48	46-H	4.71	48-H	4.00
46-H	1.59	47-H	4.34	46-H	1.74	47-H	1.71	49-H	4.06
47-H	1.05	48-H	5.99	47-H	1.63	49-H	2.28	50-H	4.08
49-H	1.59	49-H	7.63	50-H	4.76	50-H	1.88	51-H	4.33
50-H	1.12			51-H	1.92	51-H	2.06	52-H	4.67
51-H	1.38			52-H	7.57	52-H	1.98	53-H	7.47
						54-H	1.66		
						55-H	7.62		

### 3.2 CO-Releasing

The commonly used method for determining the CO-releasing of a molecule is UV–visible spectroscopy.^64^ There are various ways for releasing CO from a molecule such as substitution, decomposition, and photoactivation.^65,66^ Manganese carbonyl complexes are known as photoactivatable CO-releasing molecules (photoCORM). A photoCORM must be stable in the dark while it releases CO upon exposure to light of a certain wavelength. To demonstrate the dark stability of the molecules, UV–visible spectra were recorded for 16 h in the dark at 30-min intervals using solutions of the complexes prepared in DMSO, and no decomposition was observed, which proves the stability of the molecules in the dark. These solutions were also exposed to UV light at a wavelength of 365 nm from a distance of 2 cm, and the changes in the absorbance values of the molecules were recorded at 1-min intervals; the results confirmed that the molecules were decomposed by this light. The basic principle of the myoglobin assay is based on the formation of carbon monoxymyoglobin with the interaction of deoxymyoglobin with CORMs by the spectrophotometric method. While deoxymyoglobin exhibits a maximum absorbance at 557 nm in the spectrum, carbonmonoxy myoglobin shows two maxima at 540 and 577 nm, and this transformation could be followed/recorded in the UV–visible spectrophotometer. A 15 μM solution of the complexes was incubated with a 60 μM myoglobin solution reduced with sodium dithionite in a pH 7.4 buffered medium, and the change was followed by the spectroscopic method. The CO-releasing properties of a molecule could be evaluated from two perspectives: the first is how fast the molecule’s release is, and the second is how high the rate of the releasing is. While **2e** allows for the formation of 40.41 μM MbCO, it can release 90% of the total CO. However, 50% of the total amount of CO of **2e** was released in 7.49 min. On the other hand, **2a** could release 77% of the total CO and could release 50% of the total CO release in only 4.02 min. According to the results, **2a** would be advantageous if a rapid CO release is needed in the tissue, while **2e** would be more suitable if more regular CO release is needed. All the CO-releasing details of the molecules are presented in Table 4.

**Table 4:** Co-Releasing Details of Manganese Carbonyl Complexes with a 365 nm Lamp

Molecules	[MbCO] (μM)	Half-life *t*_1/2_ [min]	Released-CO Equivalent	Released-CO Percent [%]
**2a**	34.64	4.02	2.30	77
**2b**	39.51	4.85	2.63	88
**2c**	39.81	3.14	2.65	88
**2d**	30.34	5.47	2.02	67
**2e**	40.41	7.49	2.69	90

### 3.3 Molecule Geometry and Thermochemistry

The first crucial step in computational studies is to determine the optimized geometry of the stationary points of related molecules for further simulations and analyses. In this regard, the computed bond lengths and angles of the optimized structures of complexes **2a**–**e** depicted in Figure 1 are summarized in Table 5.

**Figure 1:**
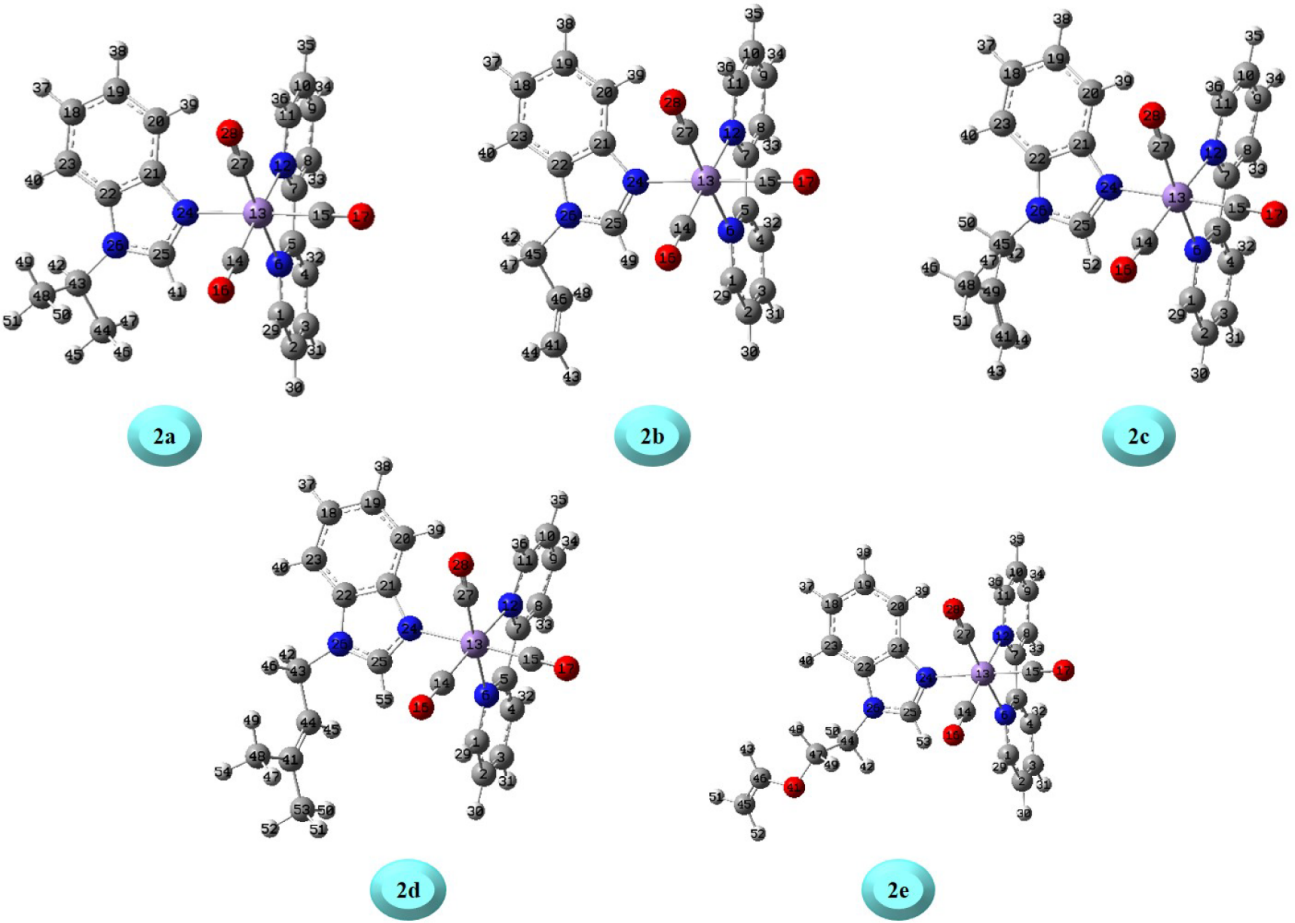
Optimized structures of compounds in the gas phase.

**Table 5:** Selected Optimized Parameters for the Complexes **2a**–**2e**

	**2a**	**2b**	**2c**	**2d**	**2e**	Exp.^a^
Bond Length (Å)						
Mn–N6	2.081	2.081	2.081	2.081	2.081	2.043
Mn–N12	2.085	2.085	2.084	2.085	2.084	2.076
Mn–N24	2.142	2.143	2.142	2.140	2.145	
C14–O16	1.143	1.143	1.143	1.143	1.143	1.151
C15–O17	1.141	1.140	1.141	1.141	1.140	1.138
C27–O28	1.142	1.142	1.142	1.142	1.142	1.146
N6–C1	1.343	1.343	1.343	1.343	1.343	
N12–C7	1.355	1.354	1.354	1.354	1.354	1.350
N24–C21	1.398	1.399	1.399	1.398	1.399	
N26–C22	1.389	1.389	1.389	1.387	1.389	
N26–C43 (-C45/-C44)	1.483	1.474	1.476	1.480	1.462	
Bond angle (deg)						
C14–Mn–C15	91.58	91.52	91.63	91.48	91.62	88.60
C14–Mn–C27	89.23	89.21	89.16	89.27	89.19	87.02
C15–Mn–C27	90.56	90.54	90.48	91.48	90.40	87.90
Mn–C14–O	179.13	179.27	179.17	179.26	179.13	175.9
Mn–C15–O	179.29	179.27	179.27	179.32	179.25	175.9
Mn–C27–O	178.23	178.23	178.13	178.48	178.08	173.1
Mn–N6–C1	125.94	125.94	125.93	125.94	125.94	126.9
Mn–N12–C7	115.41	115.42	115.41	115.40	115.41	115.9
Mn–N24–C21	131.55	131.57	131.63	131.57	131.62	
N26–C43–C44	111.44	—	—	113.10	—	
N26–C45–C46	—	112.62	—	—	—	
N26–C45–C49	—	—	112.68	—	—	
N26–C44–C47	—	—	—	—	112.65	

a Experimental data are taken from ref 67.

The Mn–N6, Mn–N12, and Mn–N24 bond lengths of complexes **2a**–**e** were estimated to be 2.81 Å, 2.084–2.085 Å, and 2.140–2.145 Å, respectively. The carbonyl group (C≡O) bond length of the complexes was computed to fall within the range of 1.140–1.143 Å. In the past, the Mn–N and C≡O distances of the organic ligand-decorated Mn­(I) tricarbonyl complexes were observed by X-ray crystallography at 2.043–2.076 Å and 1.38–1.51 Å, respectively.^67^ For complexes **2a**–**e**, the carbonyl groups’ angles chelated to the central Mn atom were calculated as C14–Mn–C15 = 91.48–91.62°, C14–Mn–C27 = 89.16–89.27°, and C15–Mn–C27 = 90.40–91.48°, respectively, with distortions of 1.48–1.62°, 0.84–0.73°, and 0.40–0.48° from the right angle (90°). Cohen and coworkers have reported the Mn–C≡O group angles for a structurally geometrically similar complex (C_16_H_9_BrMnN_3_O_3_) in the range of 173.1–175.9°, respectively.^67^ Herein, the Mn–N6–C1, Mn–N12–C7, and Mn–N24–C21 angles of **2a** were calculated to be 125.94°, 115.41°, and 131.55°, respectively, whereas these angles for **2c** were determined to be 125.93°, 115.41°, and 131.63°. On the other hand, the Mn–N6–C1 and Mn–N12–C7 bond angles of the structurally similar complex have been observed at 129.9° and 115.9°, respectively.

As is well known, thermodynamic characterizations of related systems have provided useful information on the balanced energetic forces directing the binding interactions between specific ligands and target sites in designing rational drug agents.^68^ Herein, the thermochemistry, dipole moment (μ), and polarizability value (α) of complexes **2a**–**e** are presented in Table 2. The Δ*E*, Δ*H*, and Δ*G* quantities of **2a** including the 1-isopropyl benzimidazole group were estimated to be −1436.978038, −1436.948821, and −1437.038434 au, respectively, whereas the **2e** complex including the 2-(vinyloxyethyl)­benzimidazole substitution was computed to have values of −1550.279519, −1550.248329, and −1550.344509 au, which were the highest values among the complexes. The lowest thermodynamic values among the complexes were determined for the **2b** complex (49 atoms and electrons); that is, the Δ*E*, Δ*H*, and Δ*G* quantities were predicted to be −1435.760696, −1435.732015, and −1435.821102 au, respectively. From Table 6, we can see E_therm._ (kcal/mol) quantities of the complexes calculated as **2d** (285.593) > **2e** (270.678) > **2c** (267.041) > **2a** (263.430) > **2b** (248.654), implying that the **2d** compound would have the highest thermal energy, and vice versa for **2b**. Moreover, the heat capacity of **2d** was determined to be the highest among the complexes, with the *C*_v_ (cal/mol·K) order of **2d** (115.797) > **2e** (113.227) > **2c** (110.350) > **2a** (107.488) > **2b** (104.865). Moreover, the entropies of the complexes were estimated in the following order: **2d** (203.125) > **2e** (202.427) > **2c** (194.997) > **2a** (188.606) > **2b** (187.499), which implied that the 2d complex would have the highest entropy, and vice versa for **2b**. It is worth recalling that the main contribution to thermodynamic quantities has been sourced from vibrational degrees of freedom, depending on quantum statistics.^69–71^ The dipole moments of the complexes (in D) were determined as **2a** (7.897)> **2b** (7.751)> **2e** (7.741) > **2c** (7.662) > **2d** (7.299): the 1-isopropyl-benzimidazole substitution made the complex to have a slightly higher dipole moment in comparison to the other complexes. On the other hand, the 3,3-dimethylallyl)­benzimidazole substitution to the core structure could cause a rise in polarizability; that is, complex **2d** could have the highest polarizability among the complexes, while the 1-allylbenzimidazole substitution could result in lower polarizability compared to the other substitutions.

**Table 6:** Thermochemical Quantities and Physical Parameters

		**2a**	**2b**	**2c**	**2d**	**2e**
B3LYP	Δ*E* (au)	–1436.978038	–1435.760696	–1475.062629	–1514.363877	–1550.279519
	Δ*H* (au)	–1436.948821	–1435.732015	–1475.032498	–1514.332151	–1550.248329
	Δ*G* (au)	–1437.038434	–1435.821102	–1475.125147	–1514.428663	–1550.344509
	*E*_therm._(kcal/mol)	263.430	248.654	267.041	285.593	270.678
	*C*_v_ (cal/mol.K)	107.488	104.865	110.350	115.797	113.227
	*S* (cal/mol.K)	188.606	187.499	194.997	203.125	202.427
	μ (D)	7.897	7.751	7.662	7.299	7.741
	α (au)	305.31	304.77	315.39	330.38	323.78
PBEPBE	Δ*E* (au)	–1435.419578	–1434.211334	–1473.457208	–1512.702345	–1548.602174
	Δ*H* (au)	–1435.390041	–1434.182329	–1473.426719	–1512.670233	–1548.570642
	Δ*G* (au)	–1435.480039	–1434.271911	–1473.519822	–1512.767404	–1548.667277
	*E*_therm._ (kcal/mol)	257.646	243.143	261.095	279.185	264.586
	*C*_v_ (cal/mol.K)	109.636	107.000	112.632	118.206	115.590
	*S* (cal/mol.K)	189.417	188.542	195.952	204.514	203.386
	μ (D)	8.422	8.303	8.085	7.814	8.106
	α (au)	323.28	322.47	333.65	349.24	344.79

### 3.4 NBO Simulations

As is well known, NBO computations provide information on bonding orbitals with electron density, resonance, anomeric, and conjugation interactions in specific molecular systems by analyzing second-order perturbation theory, and they are commonly applied to molecular systems.^72,73^

For complex **2a**, the electron donation from LP (2) N12 (ED_i_ = 0.77533) to the antibonding molecular orbitals of π* C7–C8 (ED_j_ = 0.16417e) and π* C10–C11 (ED_j_ = 0.13750e) would be responsible for *E* ^(2)^ values of 24.52 and 23.99 kcal/mol, respectively, with remarkable electron density on the unfilled molecular orbitals. Also, LP (1) N26 (ED_i_ = 0.63572) → π* C22–C23 (ED_j_ = 0.21998e) resonance interaction was determined with an *E* ^(2)^ value of 19.44 kcal/mol. Also, the resonance interactions of Mn → π* C15–O17 (*E* ^(2)^= 8.05 kcal/mol), LP (2) Mn → π* C14–O16 (*E* ^(2)^= 6.06 kcal/mol), and LP (2) Mn → π* C15–O17 (*E* ^(2)^= 7.05 kcal/mol) would contribute to lowering the stabilization energy significantly. On the other hand, the d → π* interactions for the other complexes (**2b**–**e**) would have greater *E* ^(2)^ than those of **2a**. The *E* ^(2)^ for the interaction LP Mn → π* C–O for complexes **2b**–**e** was calculated in the ranges of 12.11–16.01, 12.15–16.05, 12.41–16.40, and 11.94–15.81 kcal/mol, respectively. For **2b**, the highest energy contributions to the *E* ^(2)^ were determined for the resonances π C3–C4 (ED_i_ = 0.77533) → π* C5–N6 (ED_j_ = 0.21998e), π C8–C9 (ED_i_ = 0.77533e) → π* C7–N12 (ED_j_ = 0.21998e), π C18–C23 (ED_i_ = 0.77533e) → π* C22–N26 (ED_j_ = 0.21998e), and π C22–N26 (ED_i_ = 1.79864e) → π* N24–C25 (ED_j_ = 0.41154e), with *E* ^(2)^ values of 32.79, 33.14, and 35.08 kcal/mol, respectively. On the other hand, the electron movement to unfilled orbitals π* C7–C8 and π* C10–C11 from LP (1) N12 for **2c** would have the highest contribution to the decreasing energy with *E* ^(2)^ of 49.06 and 47.98 kcal/mol. The energies of counterpart interactions (LP N → π* C–C) for **2e** were calculated to be 48.96 and 47.91 kcal/mol. Moreover, the *E* ^(2)^ values of LP (1) N6 → LP* (5) Mn and LP (1) N24 → LP* (5) Mn interactions for **2c** were predicted to be 17.87 and 16.36 kcal/mol, respectively. The corresponding interaction (LP (1) N6 → LP* Mn) for **2d** and **2e** was determined with *E* ^(2)^ of 15.36–31.60 and 15.00–18.25 kcal/mol, respectively (Table 7).

**Table 7:** NBO Analysis Results

Donor(i)	ED_i_/e	Acceptor (j)	ED_j_/e	*E*^(2)^/kcal mol^–1^	*E*(j) – *E*(i)/a.u	*F*(i.j)/a.u
**2a**						
π C1–C2	0.80913	π* C3–C4	0.14030	10.75	0.29	0.072
π C3–C4	0.80833	π* C5–N6	0.24569	16.39	0.24	0.082
π C5–N6	0.86482	π* C1–C2	0.14000	11.62	0.34	0.080
π C7–C8	0.80436	π* C5–N6	0.24569	10.01	0.24	0.064
π C18–C19	0.80469	π* C20–C21	0.21904	11.09	0.27	0.070
		π* C22–C23	0.21998	10.54	0.27	0.068
π C20–C21	0.80531	π* C18–C19	0.18810	10.38	0.29	0.069
		π* C22–C23	0.21998	10.37	0.28	0.069
π C22–C23	0.81374	π* C18–C19	0.18810	10.44	0.30	0.070
		π* C20–C21	0.21904	9.37	0.29	0.067
LP (2) N12	0.63572	π* C7–C8	0.16417	24.52	0.24	0.109
		π* C10–C11	0.13750	23.99	0.24	0.110
LP (1) N6	0.82470	LP* (5) Mn	0.11610	9.40	0.56	0.095
LP (1) Mn	0.87964	π* C15–O17	0.06453	8.05	0.26	0.060
LP (2) Mn	0.87096	π* C14–O16	0.06421	6.06	0.32	0.058
		π* C15–O17	0.06453	7.05	0.27	0.057
LP (1) N26	0.77533	π* C22–C23	0.21998	19.44	0.30	0.097
**2b**						
π C1–C2	1.61647	π* C3–C4	0.28145	21.58	0.29	0.073
		π* C5–N6	0.49215	16.36	0.25	0.058
π C3–C4	1.61665	π* C1–C2	0.27947	17.69	0.28	0.064
		π* C5–N6	0.49215	32.79	0.24	0.082
π C5–N6	1.72969	π* C1–C2	0.27947	23.58	0.34	0.080
		π* C3–C4	0.28145	11.36	0.35	0.056
		π* C7–N12	0.49781	10.62	0.31	0.054
π C7–N12	1.72936	π* C5–N6	0.49215	10.92	0.30	0.054
		π* C8–C9	0.28156	11.23	0.35	0.056
		π* C10–C11	0.27496	23.48	0.35	0.081
π C8–C9	1.61469	π* C7–N12	0.49781	33.14	0.24	0.082
		π* C10–C11	0.27496	17.28	0.28	0.064
π C10–C11	1.60785	π* C7–N12	0.49781	17.08	0.24	0.059
		π* C8–C9	0.28156	21.51	0.29	0.072
π C18–C23	1.71047	π* C19–C20	0.29708	18.77	0.29	0.066
		π* C22–N26	0.79659	35.08	0.21	0.088
π C19–C20	1.71322	π* C18–C23	0.30252	19.52	0.29	0.067
π C22–N26	1.79864	π* N24–C25	0.41154	33.65	0.32	0.097
LP (1) Mn	1.75943	π* C15–O17	0.12904	16.01	0.26	0.060
LP (2) Mn	1.74241	π* C14–O16	0.12793	12.11	0.32	0.057
		π* C27–O28	0.13331	14.29	0.27	0.057
LP (1) N24	1.65145	LP* (5) Mn	0.23186	17.29	0.55	0.090
**2c**						
π C1–C2	1.61659	π* C3–C4	0.28110	21.56	0.29	0.073
		π* C5–N6	0.49235	16.36	0.25	0.058
π C3–C4	1.61635	π* C1–C2	0.27938	17.64	0.28	0.064
		π* C5–N6	0.49235	32.84	0.24	0.082
π C5–N6	1.72995	π* C1–C2	0.27938	23.20	0.35	0.080
		π* C3–C4	0.28110	11.12	0.35	0.056
		π* C7–C8	0.32832	9.73	0.31	0.053
π C7–C8	1.60864	π* C5–N6	0.49235	19.99	0.24	0.064
π C18–C23	1.71153	π* C19–C20	0.29706	18.72	0.29	0.066
		π* C22–N26	0.79435	35.01	0.21	0.088
π C19–C20	1.71416	π* C18–C23	0.30225	19.51	0.29	0.067
π C22–N26	1.79931	π* N24–C25	0.41126	33.37	0.32	0.097
LP (1) N6	1.64903	LP* (5) Mn	0.23212	17.87	0.56	0.092
LP (1) N12	1.65212	π* C7–C8	0.32832	49.06	0.24	0.109
		π* C10–C11	0.27498	47.98	0.24	0.110
LP (1) Mn	1.75942	π* C15–O17	0.12899	16.05	0.26	0.060
LP (2) Mn	1.74182	π* C14–O16	0.12884	12.15	0.32	0.057
		π* C27–O28	0.13349	14.49	0.27	0.058
LP (1) N24	1.65116	LP* (5) Mn	0.23212	16.36	0.55	0.087
**2d**						
π C1–N6	1.75802	π* C2–C3	0.29452	10.73	0.35	0.055
		π* C4–C5	0.32691	22.41	0.35	0.080
π C2–C3	1.60157	π* C1–N6	0.43271	33.87	0.24	0.081
		π* C4–C5	0.32691	18.55	0.28	0.066
π C4–C5	1.60235	π* C1–N6	0.43271	17.38	0.24	0.059
		π* C2–C3	0.29452	21.05	0.29	0.072
		π* C7–N12	0.49716	19.35	0.25	0.064
π C7–N12	1.72915	π* C4–C5	0.32691	10.14	0.34	0.053
		π* C8–C9	0.28156	11.23	0.35	0.056
		π* C10–C11	0.27515	23.51	0.35	0.081
π C8–C9	1.61517	π* C7–N12	0.49716	33.72	0.24	0.082
		π* C10–C11	0.27515	17.01	0.28	0.064
π C10–C11	1.60835	π* C7–N12	0.49716	17.04	0.24	0.059
		π* C8–C19	0.28156	21.51	0.29	0.072
π C18–C23	1.71085	π* C19–C20	0.29945	18.04	0.29	0.066
		π* C22–N26	0.79333	34.51	0.21	0.088
π C19–C20	1.71416	π* C18–C23	0.30403	19.43	0.29	0.067
π C22–N26	1.79416	π* N24–C25	0.41992	34.06	0.32	0.098
LP (1) N6	1.64921	LP* (5) Mn	0.23213	20.25	0.56	0.098
LP (1) N12	1.65250	LP* (5) Mn	0.23213	31.60	0.55	0.122
LP (1) Mn	1.75841	π* C15–O17	0.12984	16.40	0.26	0.060
LP (2) Mn	1.74158	π* C14–O16	0.12878	12.41	0.32	0.058
		π* C27–O28	0.13334	13.54	0.27	0.056
LP (1) N24	1.64781	LP* (5) Mn	0.23213	15.36	0.55	0.085
**2e**						
π C1–C2	1.61789	π* C3–C4	0.27961	21.45	0.29	0.072
		π* C5–N6	0.49277	16.33	0.25	0.058
π C3–C4	1.61532	π* C1–C2	0.27938	17.74	0.28	0.064
		π* C5-N6	0.49277	32.97	0.24	0.082
π C5–N6	1.73077	π* C1–C2	0.27938	23.14	0.34	0.080
		π* C3–C4	0.27961	11.07	0.35	0.056
		π* C7–C8	0.32804	9.70	0.35	0.053
π C7–C8	1.60872	π* C5–N6	0.49277	20.06	0.24	0.064
π C18–C23	1.71093	π* C19–C20	0.29451	18.64	0.29	0.066
		π* C22–N26	0.79996	35.35	0.20	0.088
π C19–C20	1.71243	π* C18–C23	0.30190	19.62	0.29	0.067
π C22–N26	1.79940	π* N24–C25	0.41302	33.24	0.32	0.097
LP (1) N6	1.64909	LP* (5) Mn	0.23208	15.00	0.56	0.084
LP (2) N12	1.27231	π* C7–C8	0.32804	48.96	0.24	0.109
		π* C10–C11	0.27486	47.91	0.24	0.110
LP (1) Mn	1.76039	π* C15–O17	0.12815	15.81	0.26	0.059
LP (2) Mn	1.74229	π* C14–O16	0.12808	11.94	0.32	0.057
		π* C27–O28	0.13299	14.82	0.27	0.059
LP (1) N24	1.65369	LP* (5) Mn	0.23208	18.25	0.55	0.092

### 3.5 FMO and MEP Analysis

Since Fukui’s published paper “A molecular theory of reactivity in aromatic hydrocarbons,” FMO investigations have provided deep insight into how molecular systems interact with each other.^74^ Until now, the FMO theory has been applied to various molecular systems to evaluate their own chemical reactivity features and elucidate the reaction mechanisms for the related reactions.^75,76^

In this work, the calculated reactivity parameters of **2a**–**e** were determined at the B3LYP level in the following orders of

**H (−I): 2e** (−8.612) > **2d** (−9.022) > **2a** (−9.097) > **2c** (−9.113) > **2b** (−9.124)

**L (−A): 2d** (−5.484) > **2c** (−5.528) > **2b** (−5.537) > **2a** (−5.539) > **2e** (−5.590)

**ΔE: 2e** (3.022) < **2d** (3.537) < **2a** (3.558) < **2c** (3.585) < **2b** (3.587)

**χ: 2e** (−7.101) < **2d** (−7.253) < **2a** (−7.318) < **2c** (−7.321) < **2b** (−7.330)

**η: 2e** (1.511) < **2d** (1.769) < **2a** (1.779) < **2c** (1.793) < **2b** (1.794)

**ω (eV): 2d** (0.547) < **2c** (0.549) < **2b** (0.550) < **2a** (0.553) < **2e** (0.613)

**ω+ (au): 2d** (0.421) < **2c** (0.423) < **2b** (0.424) < **2a** (0.427) < **2e** (0.490)

**ω- (au): 2d** (0.688) < **2c** (0.692) < **2b** (0.693) < **2a** (0.696) < **2e** (0.751)

**Δ** *N*_**max**_ **: 2c** (4.084) < **2b** (4.087) < **2d** (4.101) < **2a** (4.113) < **2e** (4.700)

**Δε**_**back‑donat.**_ **: 2b** (−0.448) = **2c** (−0.448) < **2a** (−0.445) < **2d** (−0.442) < **2e** (−0.378)

According to the B3LYP-level computations, **2b** would prefer to interact with the external molecular system with an energy gap value of Δ*E* = 3.587 eV, while the intramolecular interactions in **2e** could be preferable over intermolecular interactions. On the other hand, the PBEPBE computations implied that the **2c** compound would have the highest energy gap (2.063 eV) and hardness index (1.032 eV). In the literature, a molecular system with a large energy gap index is reported as a hard and less polarizable system.^77–81^ Bhavya and coworkers have investigated the chemical stability of 2-methoxy-5-fluoro phenyl boronic acid using the B3LYP/6-31++g­(d,p) level and suggested that this ligand could be stable because it has an energy gap value greater than 5 eV.^82^ Also, Melavanki and coworkers have reported two biologically active indole derivatives in the framework of chromen using B3LYP/6–311+G­(d,p); they have predicted that the 3-[2-oxo-2-(2-oxo-2H-chromen-3-yl)-ethylidene]-1,3-dihydro-indol-2-one (3OCE) compound could be softer than 3-[2-oxo-2-(3-oxo-3H-benzo­[f]­chromen-2-yl)-ethylidene]-1,3-dihydro-indol-2-one (3OBC) due to having a higher energy gap value.^83^ Herein, the **2b** (Δ*E* = 3.587 eV) complex would be the hardest one among the complexes because of having the highest energy gap value and vice versa for **2e**. Moreover, **2b** (χ= −7.330 eV) could be the most electronically stable complex among the complexes, while **2e** (χ = −7.101 eV) would be less stable at the B3LYP level as well as at the PBEPBE level. From Table 8, **2e** was estimated as the soft complex with an η value of 1.511 eV, while **2b** was determined as the hardest one with η = 1.794 eV, among the complexes, at the B3LYP level. Furthermore, the **2e** complex would exhibit more electrophilicity than the other complexes, and vice versa for **2d,** at the B3LYP level. The electron-donating capability of all Mn­(I) complexes would be higher than their electron-accepting capability at the B3LYP level. Also, the **2e** complex would be less stabilized via back-donation with Δε_back‑donat._ of −0.378 eV, while **2b** and **2c** (Δε_back‑donat._= −0.445 eV) would gain the highest stability among the complexes; a similar trend was predicted at the PBEPBE level. Finally, **2e** could have charge transfer capability with a Δ*N*_max_ value of 4.700 eV, more than those of the other complexes, while **2c** (Δ*N*_max_ = 4.084 eV) could be less capable of charge transfer. From Table 8, the PBEPBE computations revealed the following orders of global reactivity indices.

**Table 8:** Global Reactivity Parameters

		**2a**	**2b**	**2c**	**2d**	**2e**
B3LYP	H (−I) (eV)	–9.097	–9.124	–9.113	–9.022	–8.612
	L (−A) (eV)	–5.539	–5.537	–5.528	–5.484	–5.590
	Δ*E* (eV)	3.558	3.587	3.585	3.537	3.022
	χ (eV)	–7.318	–7.330	–7.321	–7.253	–7.101
	η (eV)	1.779	1.794	1.793	1.769	1.511
	ω (eV)	0.553	0.550	0.549	0.547	0.613
	ω^+^ (au)	0.427	0.424	0.423	0.421	0.490
	ω^–^ (au)	0.696	0.693	0.692	0.688	0.751
	Δ*N*_max_ (eV)	4.113	4.087	4.084	4.101	4.700
	Δε_back‑donat._ (eV)	–0.445	–0.448	–0.448	–0.442	–0.378
PBEPBE	H (−I) (eV)	–8.329	–8.350	–8.339	–8.255	–7.686
	L (−A) (eV)	–6.289	–6.290	–6.276	–6.233	–6.340
	Δ*E* (eV)	2.040	2.061	2.063	2.022	1.346
	χ (eV)	–7.309	–7.320	–7.308	–7.244	–7.013
	η (eV)	1.020	1.030	1.032	1.011	0.673
	ω (eV)	0.962	0.956	0.951	0.954	1.343
	ω^+^ (au)	0.833	0.826	0.822	0.825	1.217
	ω^–^ (au)	1.101	1.095	1.090	1.092	1.475
	Δ*N*_max_ (eV)	7.165	7.104	7.084	7.166	10.423
	Δε_back‑donat._ (eV)	–0.255	–0.258	–0.258	–0.253	–0.168

**H (−I): 2e** (−7.686) > **2d** (−8.255) > **2a** (−8.329) > **2c** (−8.339) > **2b** (−8.350)

**L (−A): 2e** (−6.340) < **2b** (−6.290) < **2a** (−6.289) < **2c** (−6.276) < **2d** (−6.233)

Δ ***E***: **2e** (1.346) < **2d** (2.022) < **2a** (2.040) < **2b** (2.061) < **2c** (2.063)

**χ: 2b** (−7.320) < **2a** (−7.309) < **2c** (−7.308) < **2d** (−7.244) < **2e** (−7.013)

**η**: **2e** (0.673) < **2d** (1.011) < **2a** (1.020) < **2b** (1.030) < **2c** (1.032)

**ω** (eV): **2c** (0.951) < **2d** (0.954) < **2b** (0.956) < **2a** (0.962) < **2e** (1.343)

ω^+^ (au): **2c** (0.822) < **2d** (0.825) < **2b** (0.826) < **2a** (0.833) < **2e** (1.217)

ω^–^ (au): **2c** (1.090) < **2d** (1.092) < **2b** (1.095) < **2a** (1.101) < **2e** (1.475)

Δ*N*_max_: **2c** (7.084) < **2b** (7.104) < **2a** (7.165) < **2d** (7.166) < **2e** (10.423)

Δε_back‑donat._: **2c** ≤ **2b** (−0.258) < **2a** (−0.255)< **2d** (−0.253) < **2e** (−0.168)

Also, Figure 2 exhibits electrophilic and nucleophilic attack sites for the complexes through the electron density on the FMOs. For all complexes, the HOMO primarily extends onto the three —C≡O groups and slightly onto the nitrogen atoms of the imidazole ring and one of the aromatic rings of bpy. On the other hand, the LUMO of all complexes is expanded onto the bpy unit and —C≡O groups chelated to the Mn­(I) atom. Herein, the substituted groups on the benzimidazole ring of the complexes could not play a role in electrophilic and nucleophilic attack reactions due to the absence of HOMO and LUMO density on them. From Figure 2, the MEP plots exhibit the electropositive region as the central Mn­(I) metal, whereas the three —C≡O units are covered by green (*V* = 0) as a marker of the neutral region for attacks. As is well known, MEP plots display the possible active regions for molecular systems by using a color scheme from red (*V* < 0) to blue (*V* > 0). Specifically, the red and blue colors show the electron-rich regions and electron-poor regions for the electrophiles and nucleophiles, respectively. Herein, the electron movement from —C≡O groups to Mn­(I), as well as back-donation for all complexes, more or less, should be responsible for the neutral attack regions.

**Figure 2:**
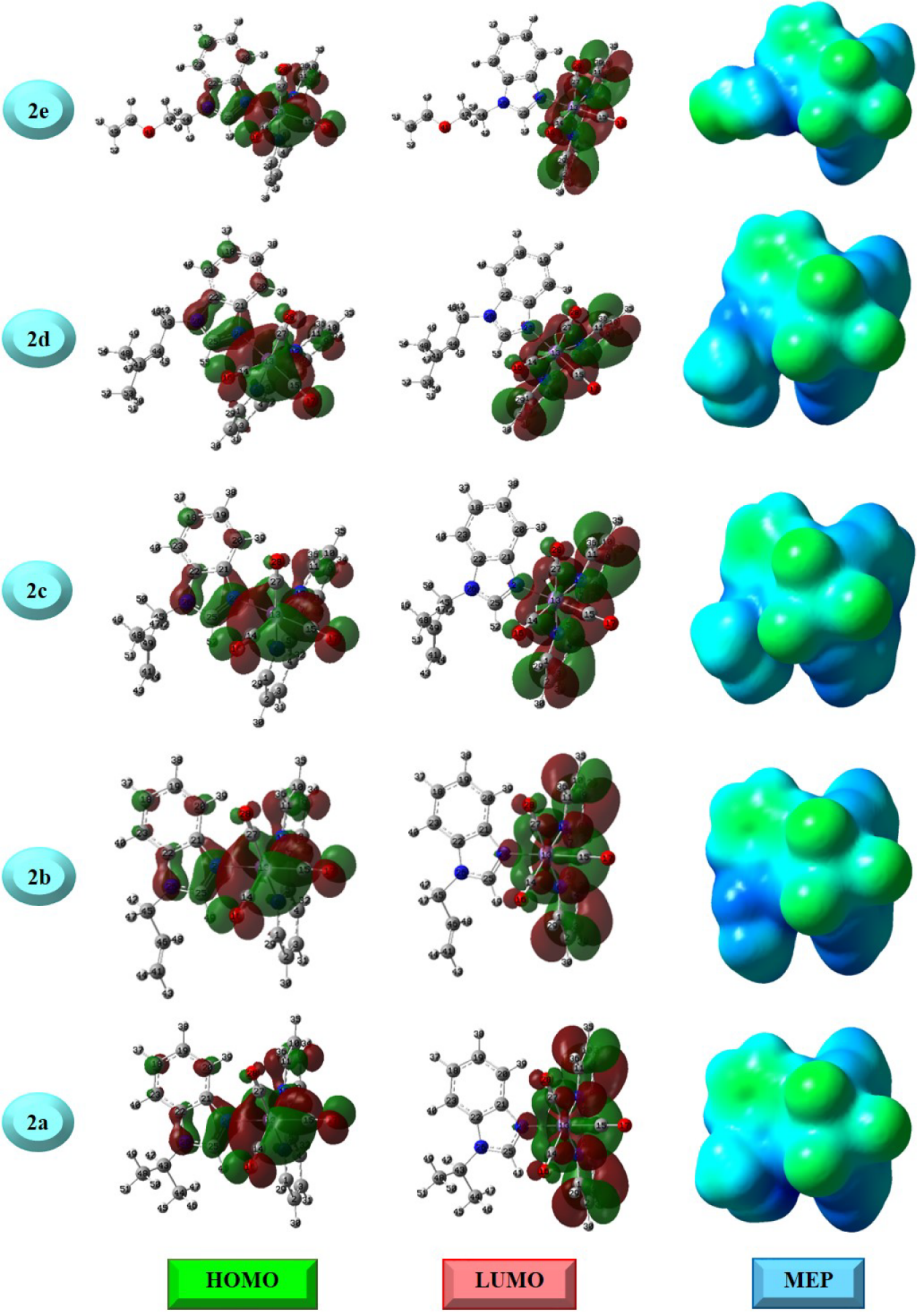
FMO amplitudes (isoval: 0.02), and MEP (isoval:0.0004) plots.

### 3.6 Molecular Docking Analysis

In recent years, developments in computational chemistry have provided useful information about the activity of molecules as well as their structural properties. The high agreement between computational and experimental methods motivates the development of new methods. One of the methods is molecular docking. In this method, the interactions of biomacromolecules with drug-candidate molecules could be evaluated both qualitatively and quantitatively. The method gives valuable data about the interaction type, region, and mechanism.^84^ Human serum albumin (HSA) is the most abundant protein in plasma and is the main carrier of endogenous and exogenous ligands including fatty acids, nucleic acids, hormones, metals, toxins, and drugs.^85^ In addition, the HSA-heme-Fe complex exhibits globin-like catalytic properties such as peroxynitrite scavenging, catalase, and peroxidase activities.^86^ HSA is also a biomarker of many diseases, including cancer, rheumatoid arthritis, ischemia, obesity, diabetes, shock, trauma, hemorrhage, acute respiratory distress syndrome, hemodialysis, acute liver failure, chronic liver disease, and hypoalbuminemia. Therefore, analyzing the interaction of a candidate molecule with human serum albumin provides insights into many issues, and in this study, the interaction of the manganese complexes with HSA was examined.^87^ According to molecular docking results, each molecule interacted with the same region of human serum albumin and formed a H-bond with Cys448. In addition to this interaction, **2a**–**d** also exhibited pi-interactions through the conjugated cyclic part with Lys195 and Lys436, and alkyl interactions with Val455 and Ala191. van der Waals interactions with Glu188, Ser192, Glu292, Pro447, Asp451, and Tyr452 could contribute to the total interaction values of these molecules. However, **2e** had additional H-bonds with Lys195 and Glu292, thanks to the oxygen in the benzimidazole derivative ligand of the molecule. The binding constants for **2a**–**e** were determined as −4.93, −4.92, −5.06, −5.18, and −5.9 kcal/mol, respectively. The higher binding constant of **2e** compared to the others can be attributed to the extra H-bonds. On the other hand, the inhibition constants of **2a**–**e** were calculated as 243.16, 243.9, 197.05, 160.11, and 67.42 μM, respectively. In this case, **2e** had the most effective connection to human serum albumin. All the interactions between the molecules and HSA are represented in Figure 3 and Table S1.

**Figure 3:**
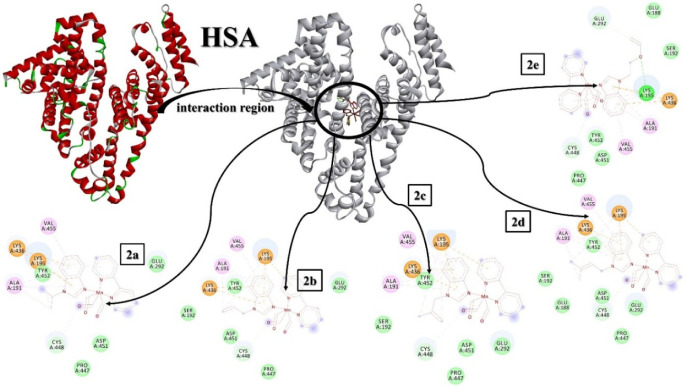
Interactions of the [Mn­(CO)_3_L­(bpy)]­PF_6_-type manganese carbonyl complexes against HSA.

The dynamic properties of BSA and HSA are different from each other, although the structures of both macromolecules are very similar. Therefore, bovine serum albumin is frequently used in experimental procedures, where serum albumin interactions are examined. The interactions of the molecules with BSA were analyzed by molecular docking methods. The binding affinity of **2a** was determined as −8.22 kcal/mol and the inhibition constant as 947.65 nM. This remarkable interaction value may be due to the H-bonds with His145, Leu189, Glu424, and Ser428. In addition, the pi interactions of **2a** with Arg458 and Ala193, alkylic interactions with Leu454, Ile455, and Arg196, and van der Waals interactions with Thr190, Ser192, and Asn457 are noteworthy. For **2b**, the binding affinity was calculated as −8.16 kcal/mol, while the inhibition constant was calculated as 1.05 μM. This molecule had an additional H-bond with Ala193, although pi interactions are relatively less than those of **2a**. All molecules interact with approximately the same region of the macromolecule. The interaction constants obtained for **2c**, **2d**, and **2e** with BSA were calculated as −7.35, −8.48, and −8.14 kcal/mol, respectively, while the inhibition constants were calculated as 4.08 μM, 604.65 nM, and 1.08 μM, respectively. All the interactions between the molecules and BSA are represented in Figure 4 and Table S1.

**Figure 4:**
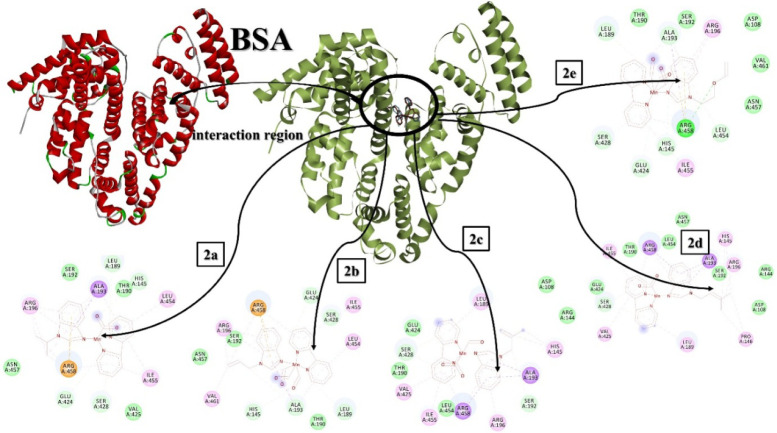
Interactions of the [Mn­(CO)_3_L­(bpy)]­PF_6_ type manganese carbonyl complexes against BSA.

Understanding drug–DNA interactions is an active area of research at the interface of chemistry, molecular biology, and medicine. The interaction of drugs with DNA is an important feature in pharmacology and plays a vital role in determining drug action mechanisms and designing more effective and specifically targeted drugs with fewer side effects. DNA is also a cellular target for many cytotoxic anticancer agents. On the other hand, the interaction between DNA and drugs can cause chemical and conformational changes and thus alter some properties of nucleobases. Additionally, the analysis of drug–DNA interactions with theoretical methods provides detailed data that cannot be obtained through experimental results. Therefore, in this study, the interactions of the characterized molecules with DNA were analyzed by molecular docking methods. All the interactions between the molecules and DNA are represented in Figure 5.

**Figure 5:**
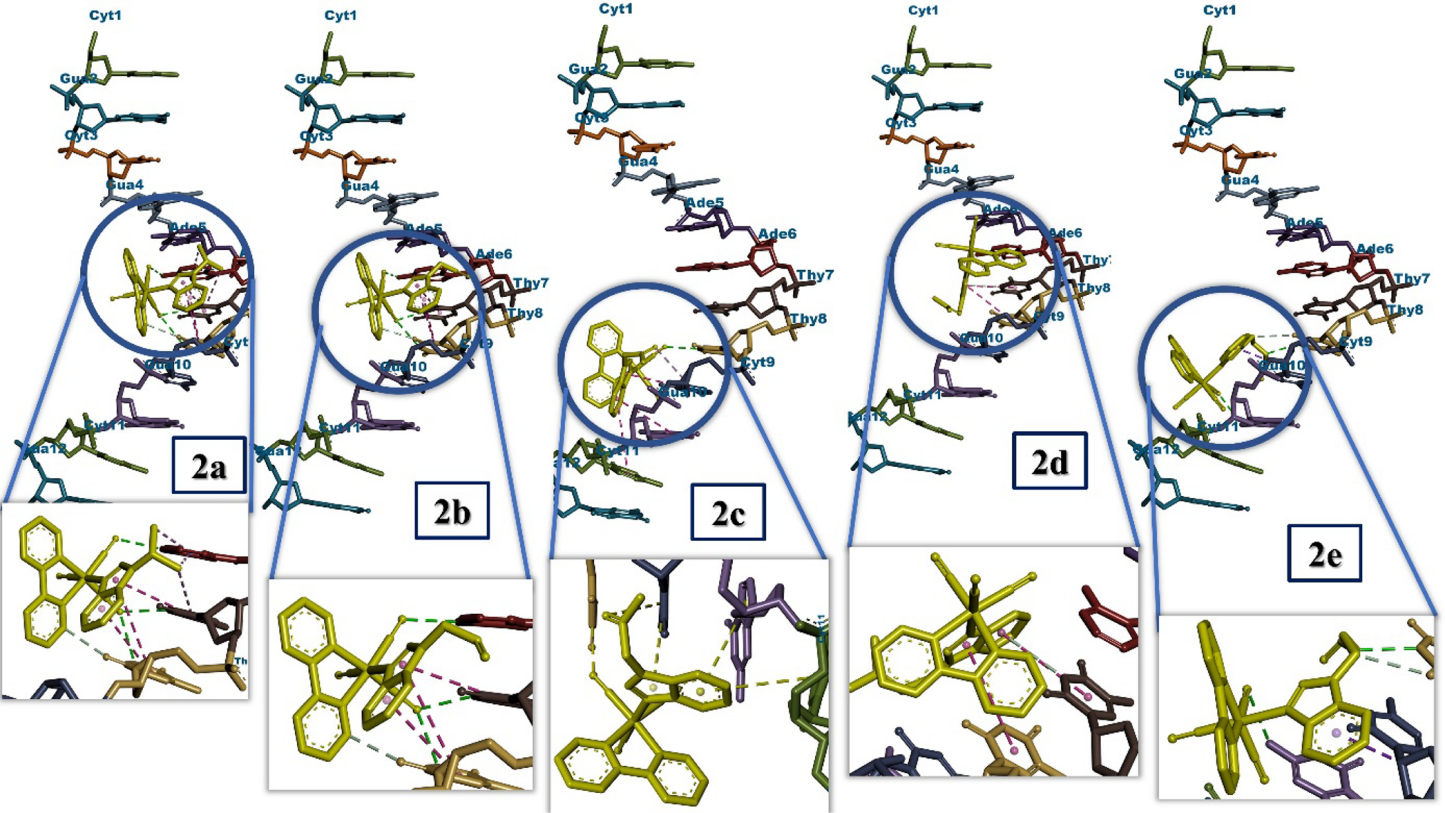
Interactions of the [Mn­(CO)_3_L­(bpy)]­PF_6_-type manganese carbonyl complexes against DNA.

### 3.7 DNA Binding Analysis

In this study, the DNA-binding affinity of the molecules was also confirmed with a UV–vis spectrophotometer. Each manganese complex exhibited a maximum at ∼388 nm. While small fluctuations for 300 μM of each complex were recorded upon the addition of DNA solutions with different concentrations (0, 20, 40, 60, 80, 100, 120, and 140 μM) at 388 nm (Figures S6–S10), remarkable changes were detected at 260 nm, which is characteristic of DNA. A constant concentration of DNA (25 μM) solution was incubated with increasing amounts of carbonyl complexes (0, 5, 10, 15, 20, 25, and 30, 35 μM), and the recorded spectra were evaluated using the Benesi–Hildebrand equation.^88,89^

$$\frac{1}{A_{\mathrm{obs}}-A_{0}}=\frac{1}{A_{c}-A_{0}}+\frac{1}{K_{b}\left(A_{c}-A_{0}\right)\left[M\right]}$$

Here, *A*_o_ is the absorbance of DNA in the absence of the manganese complex, *A*_obs_ is the absorbance of DNA at 260 nm with different amounts of the molecules, [M] is the molar concentration of the molecules, and *K*_b_ is the binding constant. The plot of 1/(*A*_obs_ – A_0_) vs 1/[M] was linear, and *K*_b_ values were calculated from the ratio of the intercept to the slope.^90^ The best binding constant was determined for **2a** as 3.2 × 10^4^ M^–1^ (*R* ^2^ : 0.9887) (Figure 6) while **2c** had a binding affinity of 1.8 × 10^4^ M^–1^ (*R* ^2^: 0.9875) (Figure S12). The DNA-binding constants of **2b**, **2d**, and **2e** were determined as 2.2 × 10^4^ M^–1^ (*R* ^2^: 0.9882) (Figure S11), 2.4 × 10^4^ M^–1^ (*R* ^2^: 0.9957) (Figure S13), and 2.7 × 10^4^ M^–1^ (*R* ^2^: 0.9867) (Figure S14), respectively. These binding constants are moderately available for DNA-target drug candidates.

**Figure 6:**
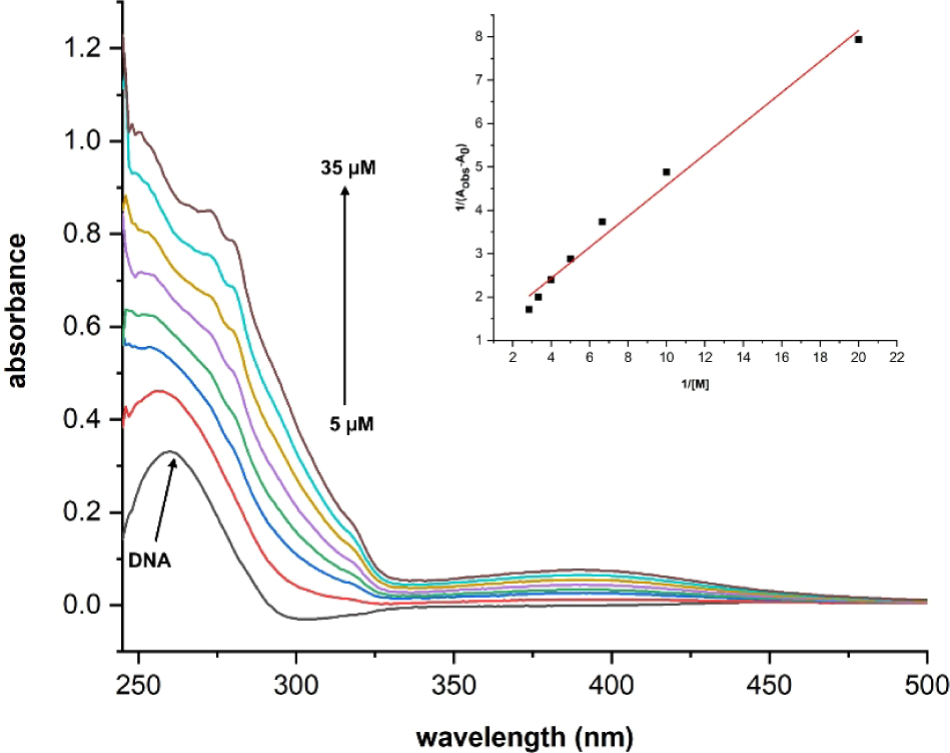
UV–vis spectra of DNA with increasing concentrations of **2a** solution (inset: the plot of 1/(*A*_obs_ – *A*_0_) vs 1/[M]).

### 3.8 BSA Binding Analysis

The BSA-binding affinity of the molecules was also confirmed with a UV–vis spectrophotometer in this study. A constant concentration of BSA (15 μM) solution was incubated with increasing concentrations of the molecules (0, 2, 4, 6, 8, and 10, 12, 14 μM), and the recorded spectra were evaluated using the Benesi–Hildebrand equation presented above, based on the identical band at 280 nm.^88,89^ The best binding constant was determined for **2d** as 8.2 × 10^4^ M^–1^ (*R* ^2^: 0.9965) (Figure 7) while **2a** exhibited a binding affinity of 3.6 × 10^4^ M^–1^ (*R* ^2^: 0.9981) (Figure S15). The DNA-binding constants of **2b**, **2c**, and **2e** were determined as 4.3 × 10^4^ M^–1^ (*R* ^2^: 0.9759) (Figure S16), 4.0 × 10^4^ M^–1^ (*R* ^2^: 0.9985) (Figure S17), and 3.9 × 10^4^ M^–1^ (*R* ^2^: 0.9998) (Figure S18), respectively. These binding constants are moderately available for BSA carrier drug candidates.

**Figure 7:**
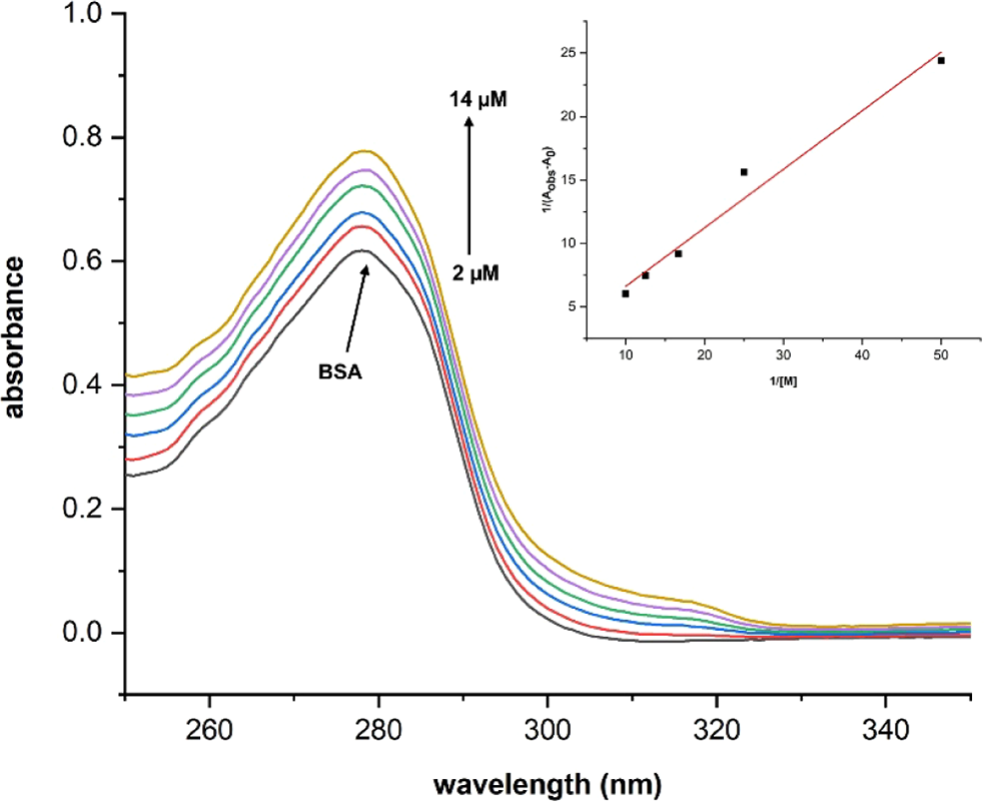
UV–vis spectra of BSA with increasing concentrations of **2d** solution (inset: the plot of 1/(*A*_obs_ – *A*_0_) vs 1/[M]).

## 4 Conclusions

[Mn­(CO)_3_(bpy)­L]­PF_6_-type metal carbonyl complexes were synthesized and characterized by FT-IR, ^1^H and ^13^C NMR, and LC-MS, and the characterization outputs were compatible with the expectations. Also, the characterization data were confirmed with DFT optimization methods. Since metal carbonyl complexes have recently been accepted as CO-releasing molecules, the compounds were evaluated for their CO-releasing properties. According to the results, **2a** would be advantageous if a rapid CO release is needed in the tissue, while **2e** would be more suitable if a more regular CO release is needed. In addition, DFT/B3LYP/6–311G­(d,p)/LANL2DZ computations disclosed that complex **2d** would have the highest thermal energy (285.593 kcal/mol), heat capacity (115.797 cal/mol·K), and entropy values (203.125 cal/mol·K). For all complexes, NBO analyses exhibited that the highest contribution to decreasing stabilization energy could be attributed to the resonance interaction through electron movement from lone pairs of nitrogen (LP N) to infilling molecular orbitals (π* C–C). The calculated η values implied that **2e** was estimated as the soft complex with an η value of 1.511 eV, while **2b** was identified as the hardest one with η = 1.794 eV among the complexes. Also, the **2e** complex was predicted to be less stabilized via back-donation with a Δε_back‑donat._ of −0.378 eV, while **2b** and **2c** (Δε_back‑donat._ = −0.445 eV) were found to gain the highest stability among the complexes. Moreover, **2e** exhibited charge transfer capability with a Δ*N*_max_ value of 4.700 eV more than the other complexes, while **2c** (Δ*N*_max_ = 4.084 eV) was the least capable of charge transfer. The molecules were also evaluated for their HSA, BSA, and DNA interaction properties by molecular docking methods. **2e** had the most effective connection with human serum albumin, while the interaction constants obtained for **2c**, **2d**, and **2e** with BSA were calculated as −7.35, −8.48, and −8.14 kcal/mol. Additionally, the interactions of the molecules with DNA were analyzed in this study with molecular docking methods. The DNA- and BSA-binding properties were evaluated by the Benesi–Hildebrand method, and, according to the results, these manganese complexes could be evaluated as moderate DNA-targeted and BSA-carrier drug candidates.

## Supplementary Material



## Notes

The synthesis procedure and the characterization details of **1a**–**e**, the recorded FT-IR, ^13^C NMR, ^1^H NMR, LC-MS spectra, and the elemental analysis report of the compounds **2a**–**e**; UV–vis spectra of DNA binding for **2b**, **2c**, **2d**, and **2e**; UV–vis spectra of BSA binding for **2a**, **2b**, **2c**, and **2e** (PDF)
