# Supplementary material for: Synthesis, Characterization, Computational Evaluation, CO-Releasing Properties, and Molecular Docking Interactions of New [Mn(CO)3(bpy)L]PF6‑Type Molecules
Source: ACS Omega. 2025 Jul 14;10(28):30798–814. doi: 10.1021/acsomega.5c03085 (PMC12290954; doi:10.1021/acsomega.5c03085)
Supplement: Supplementary file 1 [file ao5c03085_si_001.pdf]

# Synthesis, Characterization, Computational Evaluation, CO-Releasing Properties, and Molecular Docking Interactions of New $[Mn(CO)_3(bpy)L]PF_6$ Type Molecules

**Sena Ceren Önbaş[1], Goncagül Serdaroğlu[2], Neslihan Şahin[3], Elvan Üstün[1,*], İsmail Özdemir[4]**

*[3]Department of Chemistry, Faculty of Art and Science, Ordu University, 52200 Ordu, Turkey,*
*[2]Math. and Sci. Edu., Faculty of Education, Cumhuriyet University, 58140, Sivas, Turkey*
*[3]Department of Science Education, Faculty of Education, Cumhuriyet University, 58140, Sivas, Turkey*
*[4]Department of Chemistry, Faculty of Science and Art, İnönü University, 44280 Malatya, Turkey*

**Corresponding Author: elvanustun77@gmail.com*

# SUPPORTING INFORMATION

**Synthesis and Characterization of Benzimidazole Type Ligands**

The certain amount of benzimidazole was added to the NAH suspension prepared in dry THF and the mixture was stirred for 1 hour. After the alkyl halide form of the ligand were put into the mixture, it heats 24 hours at 60 $^{o}$C. After THF was evaporated under vacuum, the residue was solute with dichloromethane and the final product was achieved by distillation. These compounds, which were previously introduced to the literature, were synthesized according to our previous studies [23-28], A solution of NaH (10 mmol) in dry THF (30 mL) was prepared, and benzimidazole (10 mmol) was then added to this solution. The mixture was stirred at room temperature for 1 hour. Following this, the corresponding alkyl halide such as isopropyl bromide, allyl bromide, methallyl chloride, 3,3-dimethylallyl bromide, or 2-chloroethyl vinyl ether (10.1 mmol) was added dropwise, and the reaction was heated at 60°C for 24 hours. After the reaction was complete, the THF was removed under reduced pressure. To the remaining solid, dichloromethane (50 mL) was added, and the mixture was filtered. The clear solution obtained was distilled, yielding the desired 1-alkylbenzimidazoles (**1a-e).**

**1-isopropylbenzimidazole, (1a)** [24]

Yield: 76%. FT-IR $\nu_{(CN)}$: 1484 cm$^{-1}$; $^{1}$H NMR (400 MHz, CDCl$_3$): δ = 8.00 (s, 1H, NC*H*N), 7.82-7.80 (m, 1H, Ar-*H*), 7.44-7.42 (m, 1H, Ar-*H*), 7.32-7.25 (m, 2H, Ar-*H*), 4.97 (s, 1H, NCH$_2$C(CH$_3$)C*H*$_2$), 4.79 (s, 1H, NCH$_2$C(CH$_3$)C*H*$_2$), 4.63 (s, 2H, NC*H*$_2$C(C H$_3$)CH$_2$), 1.60 and 1.62 (s, 6H, NCH(C*H*$_3$)$_2$), $^{13}$C{$^{1}$H} NMR (100 MHz, CDCl$_3$): δ = 144.0 (N*C*HN), 140.2, 122.7, 122.3, 122.1, 120.3, 110.1 (Ar-*C*), 47.7 N*C*H(CH$_3$)$_2$, 22.6 (NCH(*C*H$_3$)$_2$).

**1-allylbenzimidazole, (1b)** [25]

Yield: 76%. FT-IR $\nu_{(CN)}$: 1615 cm$^{-1}$; $^{1}$H NMR (400 MHz, CDCl$_3$): δ = 7.93 (s, 1H, NC*H*N), 7.84 (t, 1H, Ar-*H*, $^{3}J$ = 4 Hz),7.40 (t, 1H, Ar-*H*, $^{3}J$ = 4 Hz), 7.32 (d, 2H, Ar-*H*, $^{3}J$ = 4 Hz), 6.03 (quint, 1H, NCH$_2$C*H*CH$_2$, $^{3}J$ = 8 Hz), 5.32 (d, 1H, NCH$_2$CHC*H*$_2$, $^{3}J$ = 8 Hz), 5.22 (d, 1H, NCH$_2$CHC*H*$_2$, $^{3}J$ = 16 Hz), 4.80 (s br, 2H, NC*H*$_2$CHCH$_2$). $^{13}$C NMR (100 MHz, CDCl$_3$): δ = 143.8 (N*C*HN), 142.9, 140.9, 133.8, 123.0, 122.2, 120.3, 118.7, 110.0 (Ar-C), 131.9 NCH$_2$*C*H*C*H$_2$, 47.4 (N*C*H$_2$CHCH$_2$).

**1-(2-metallyl)benzimidazole, (1c)** [26]

Yield: 78%. FT-IR $\nu_{(CN)}$: 1615 cm$^{-1}$; $^{1}$H NMR (400 MHz, CDCl$_3$): δ = 7.92 (s, 1H, NC*H*N), 7.84 (t, 1H, Ar-*H*, $^{3}J$ = 4 Hz), 7.39 (t, 1H, Ar-*H*, $^{3}J$ = 4 Hz), 7.31 (d, 2H, Ar-*H*, $^{3}J$ = 4 Hz), 5.02 (s, 1H, NCH$_2$C(CH$_3$)C*H*$_2$), 4.85 (s, 1H, NCH$_2$C(CH$_3$)C*H*$_2$), 4.71 (s, 2H, NC*H*$_2$C(CH$_3$)CH$_2$), 1.73 (s, 2H, NCH$_2$C(C*H*$_3$)CH$_2$). $^{13}$C NMR (100 MHz, CDCl$_3$): δ = 143.8 (N*C*HN), 143.3, 139.5, 134.0, 123.0, 120.4, 114.0, 110.1 (Ar-*C*), 122.2 (NCH$_2$*C*(CH$_3$)*C*H$_2$), 51.11 (N*C*H$_2$C(CH$_3$)CH$_2$), 19.8 NCH$_2$C(*C*H$_3$)CH$_2$).

**1-(3,3-dimethylallyl)benzimidazole, (1d)** [27]

Yield: 74%. FT-IR $\nu_{(CN)}$: 1613 cm$^{-1}$; $^{1}$H NMR (400 MHz, CDCl$_3$): δ = 7.87 (s, 1H, NC*H*N), 7.81-7.77 (m, 1H, Ar-*H*), 7.35-7.32 (m, 1H, Ar-*H*), 7.26-7.22 (m, 2H, Ar-*H*), 5.37-5.33 (m, 1H, NCH$_2$C*H*C(CH$_3$)$_2$), 4.68-4.65 (m, 2H, NC*H*$_2$CHC(CH$_3$)$_2$), 1.90 (s, 3H, NCH$_2$CHC(C*H*$_3$)$_2$), 1.76 (s, 3H, NCH$_2$CHC(C*H*$_3$)$_2$. $^{13}$C NMR (100 MHz, CDCl$_3$):

δ = 143.9 (N*C*HN), 133.8 ($NCH_2$*C*HC($CH_3$)$_2$), 142.5, 142.3, 138.2, 122.7, 122.2, 122.0, 120.1, 118.2, 109.9 (Ar-*C*), 42.9 (N*C*$H_2$CHC($CH_3$)$_2$), 18.1 $NCH_2$CHC(*C*$H_3$)$_2$).

**1-(2-vinyloxyethyl)benzimidazole, (1e)** [28]

Yield: 79%. FT-IR $v_{(CN)}$: 1611 $cm^{-1}$; $^1$H NMR (400 MHz, $CDCl_3$): δ = 7.99 (s, 1H, NC*H*N), 7.84 (d, 1H, Ar-*H*, $^3J$ = 8 Hz), 7.44 (d, 1H, Ar-*H*, $^3J$ = 8 Hz), 7.34-7.29 (m, 2H, Ar-*H*), 6.42 (dd, 1H, OC*H*$CH_2$, $^3J$ = 8 Hz, $^2J$ = 8 Hz), 4.45 (t, 2H, NC$H_2$$CH_2$O, $^3J$ = 4 Hz), 4.19 (d, 1H, OCHC$H_2$, $^3J$ = 16 Hz), 4.06-4.03 (m, 3H, OCHC$H_2$, $NCH_2$C$H_2$O). $^{13}$C NMR (100 MHz, $CDCl_3$): δ = 150.9 (O*C*H$CH_2$), 143.7 (N*C*HN), 133.7, 123.1, 122.3, 120.4, 115.6, 109.5 (Ar-*C*), 87.6 ($NCH_2CH_2$OCH*C*$H_2$), 65.8 ($NCH_2$*C*$H_2$OCH$CH_2$), 44.2 (N*C*$H_2CH_2$OCH$CH_2$).

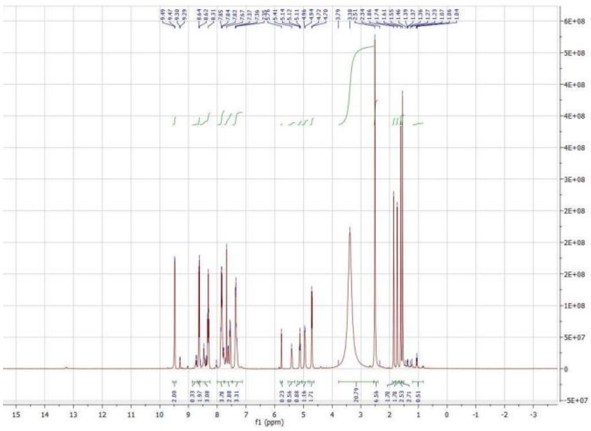

(a)

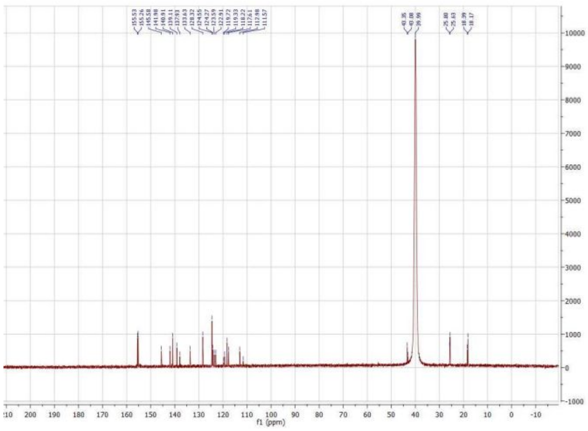

(b)

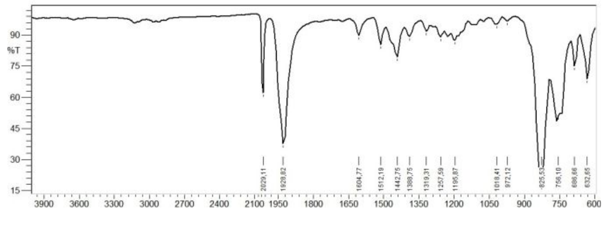

(c)

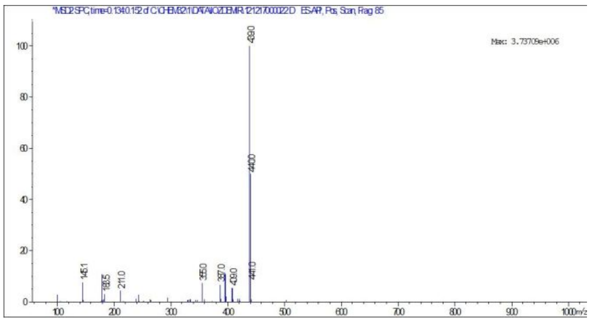

(d)

**Figure S1.** $^1$H NMR (a), $^{13}$C NMR (b), FT-IR (c) and LC-MS (d) spectra of **2a**

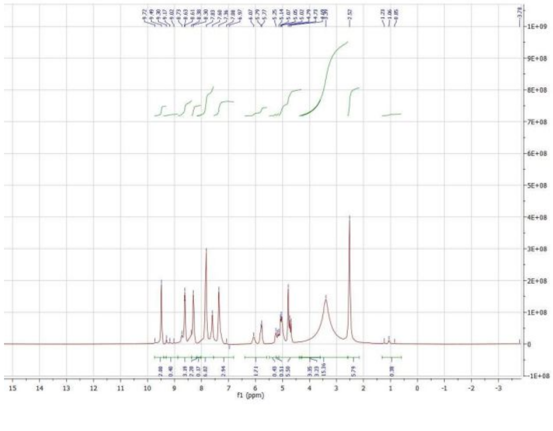

(a)

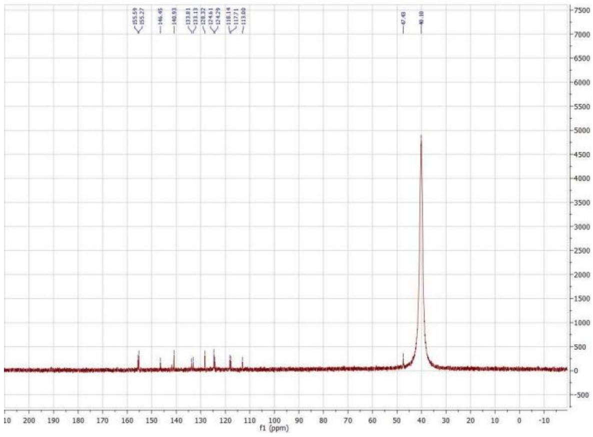

(b)

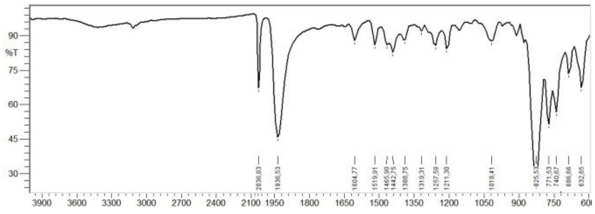


(c)

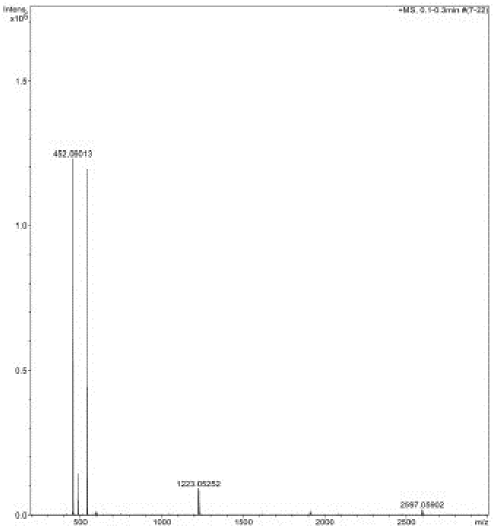


(d)

**Figure S2.** $^{1}$H NMR (a), $^{13}$C NMR (b), FT-IR (c) and LC-MS (d) spectra of **2b**

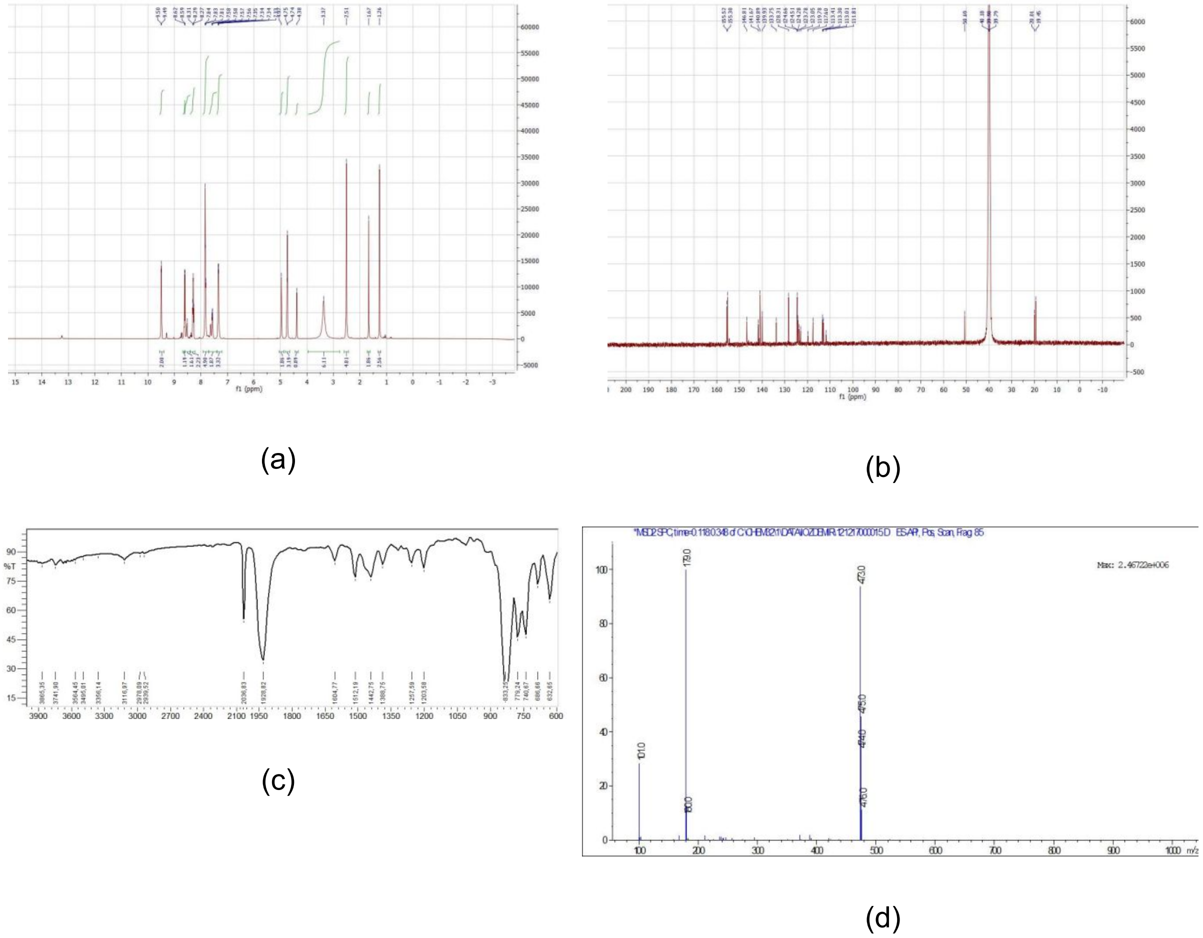


**Figure S3.** $^{1}H$ NMR (a), $^{13}C$ NMR (b), FT-IR (c) and LC-MS (d) spectra of **2c**

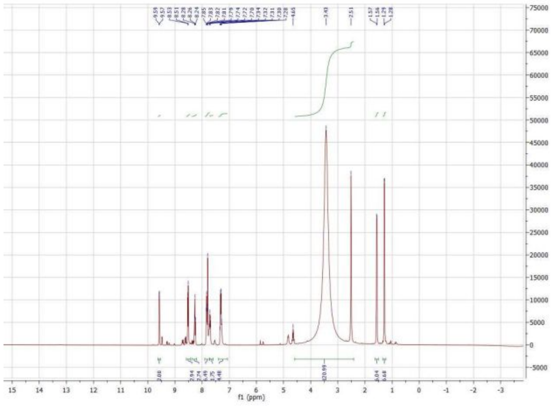


(a)

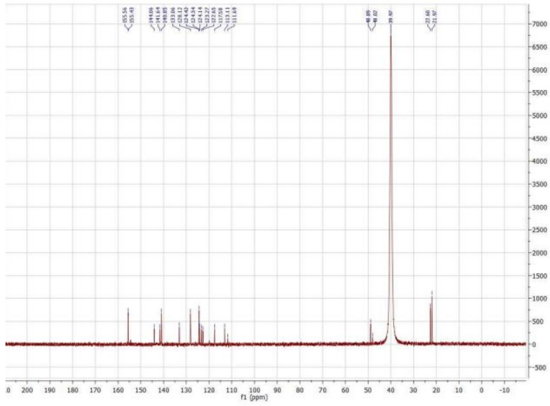


(b)

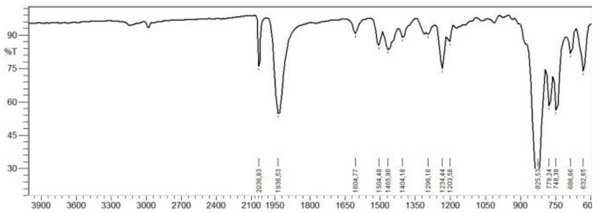


(c)

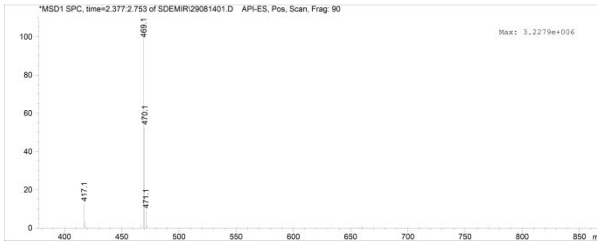


(d)

**Figure S4.** $^{1}$H NMR (a), $^{13}$C NMR (b), FT-IR (c) and LC-MS (d) spectra of **2d**

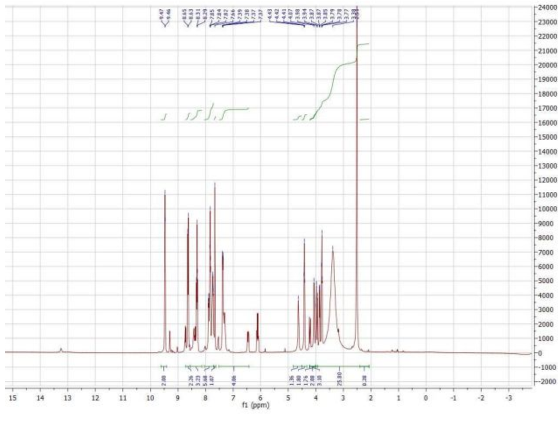

(a)

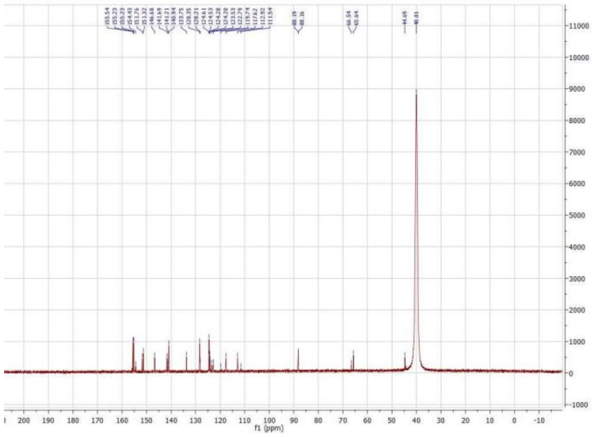

(b)

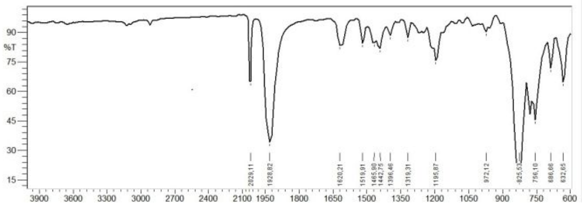

(c)

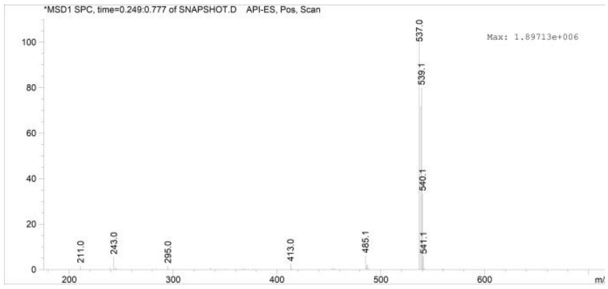


(d)

**Figure S5.** $^{1}$H NMR (a), $^{13}$C NMR (b), FT-IR (c) and LC-MS (d) spectra of **2e**

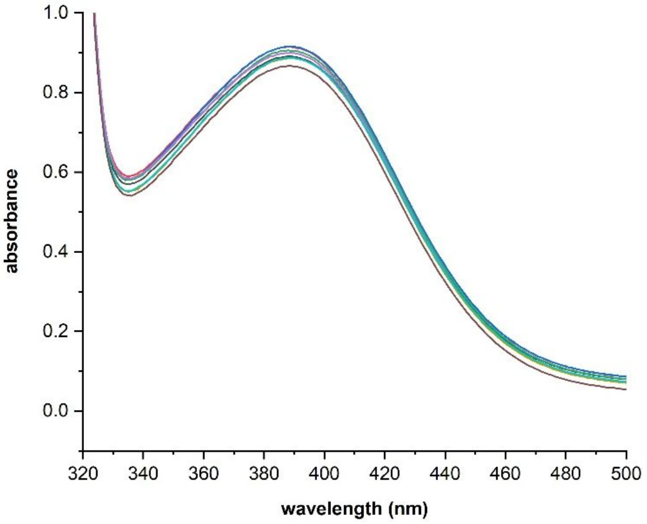


**Figure S6.** The UV-Vis spectra of **2a** with increasing concentrations of DNA solution

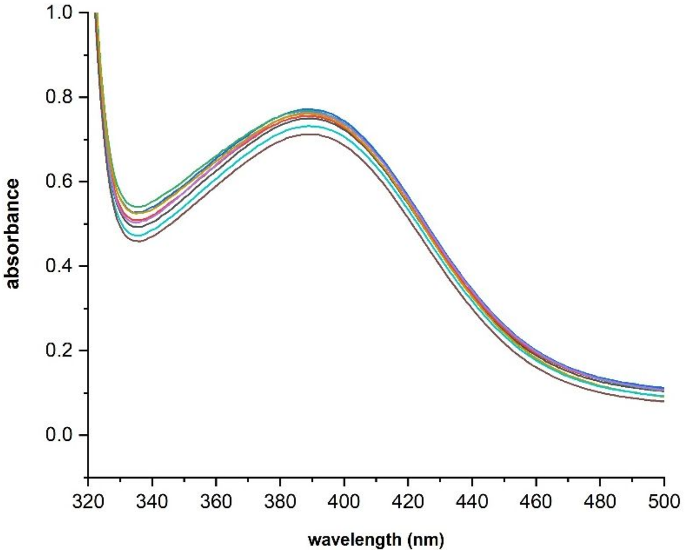


**Figure S7.** The UV-Vis spectra of **2b** with increasing concentrations of DNA solution

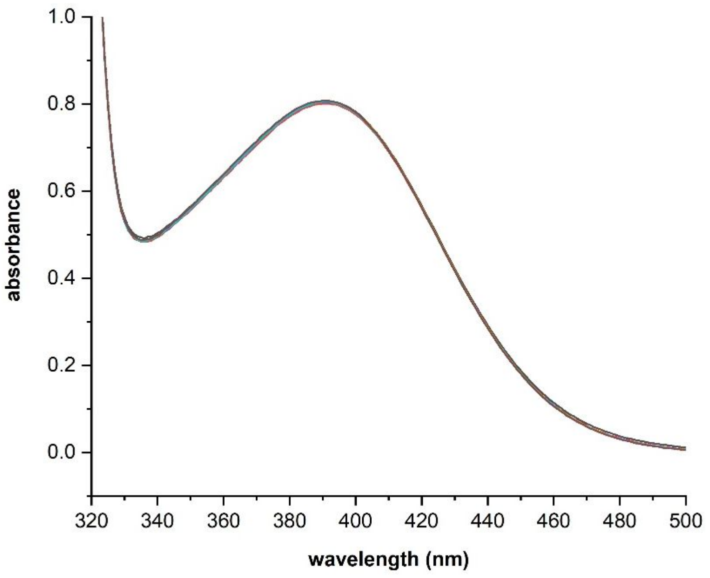


**Figure S8.** The UV-Vis spectra of **2c** with increasing concentrations of DNA solution

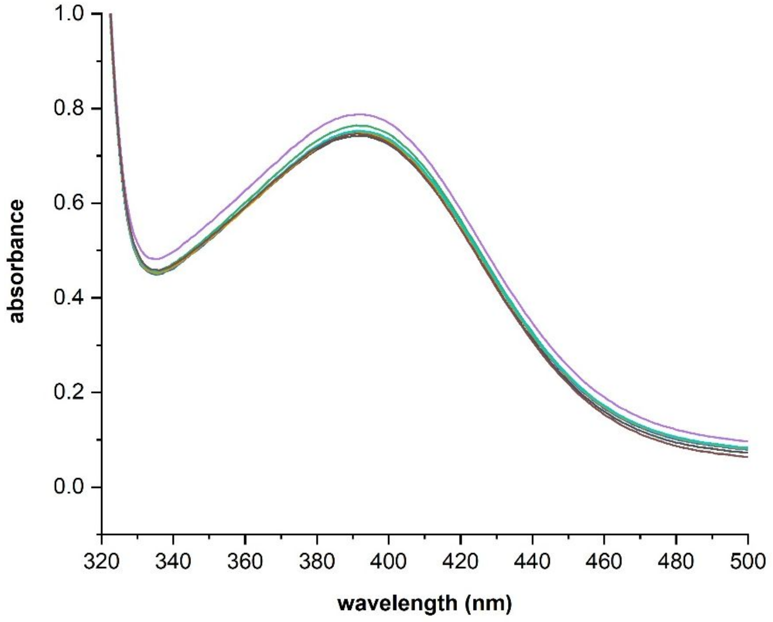


**Figure S9.** The UV-Vis spectra of **2d** with increasing concentrations of DNA solution

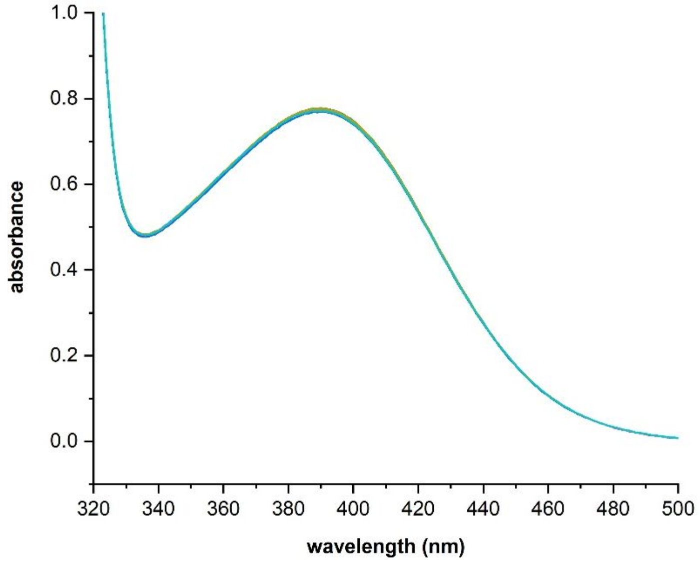


**Figure S10.** The UV-Vis spectra of **2e** with increasing concentrations of DNA solution

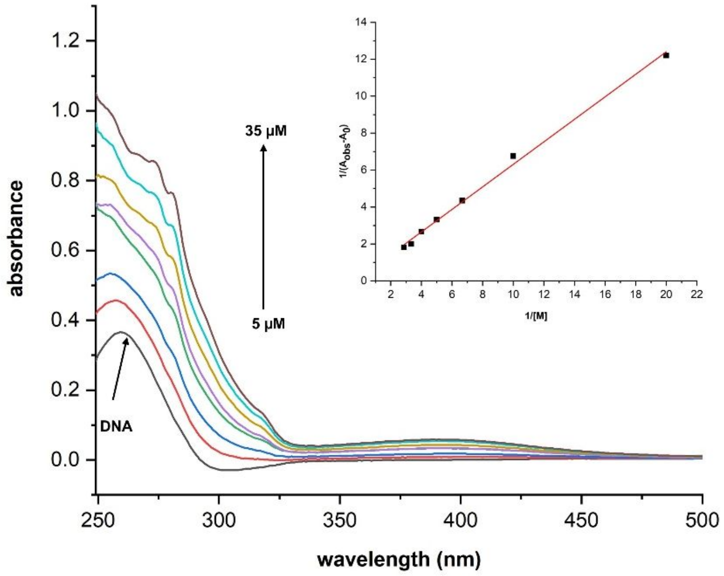


**Figure S11.** The UV-Vis spectra of DNA with increasing concentrations of **2b** solution

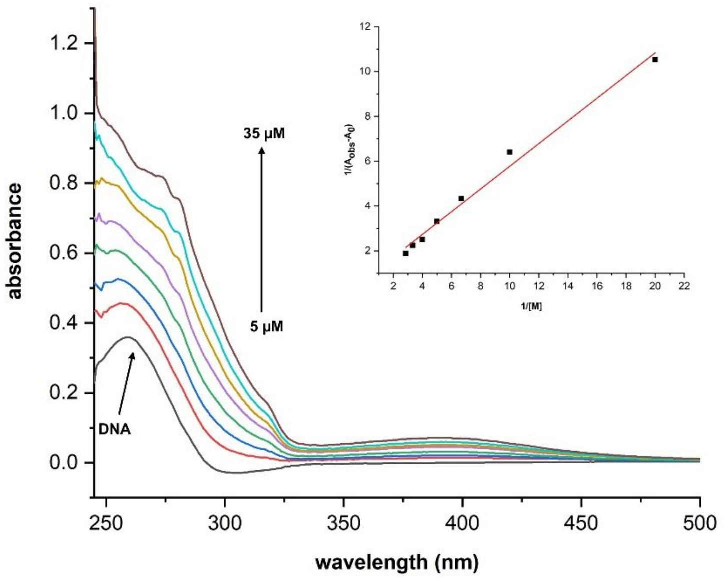


**Figure S12.** The UV-Vis spectra of DNA with increasing concentrations of **2c** solution

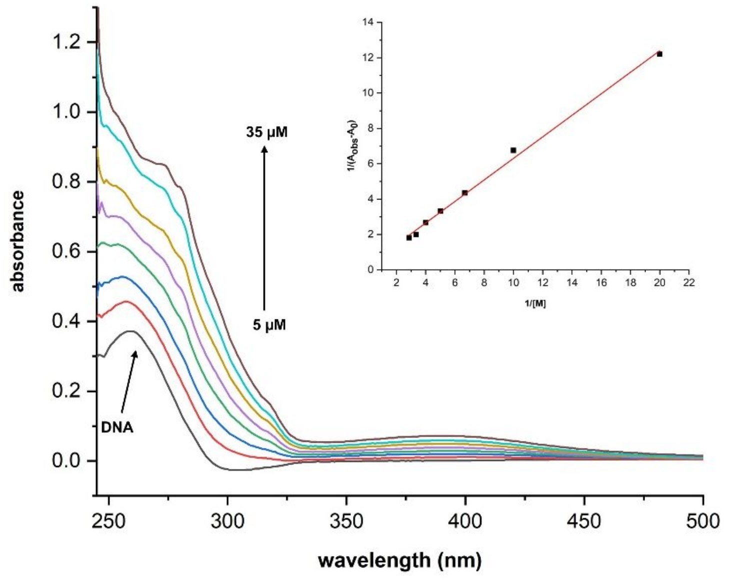


**Figure S13.** The UV-Vis spectra of DNA with increasing concentrations of **2d** solution

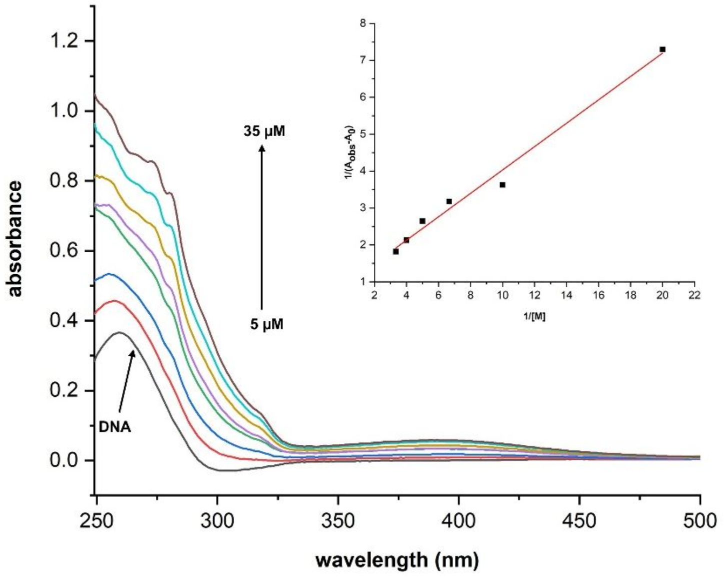


**Figure S14.** The UV-Vis spectra of DNA with increasing concentrations of **2e** solution

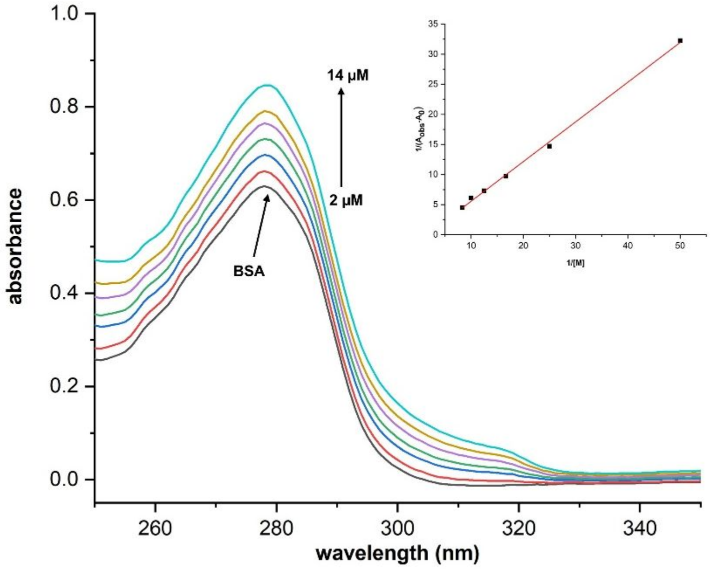


**Figure S15.** The UV-Vis spectra of BSA with increasing concentrations of **2a** solution

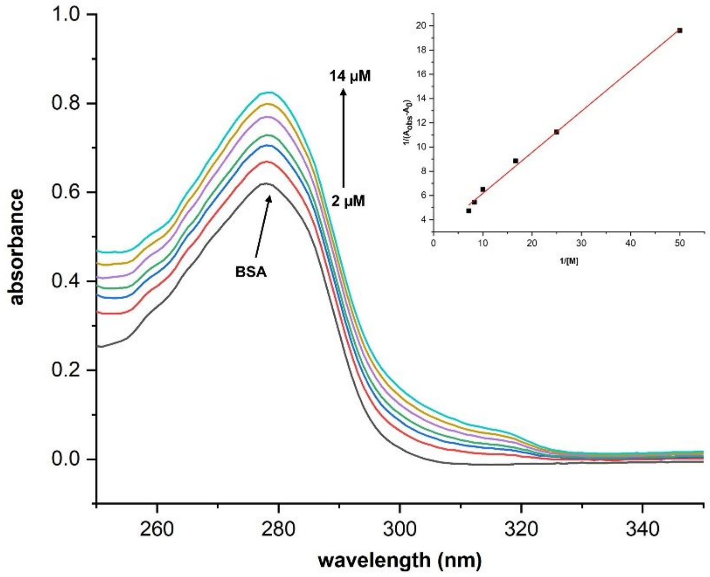


**Figure S16.** The UV-Vis spectra of BSA with increasing concentrations of **2b** solution

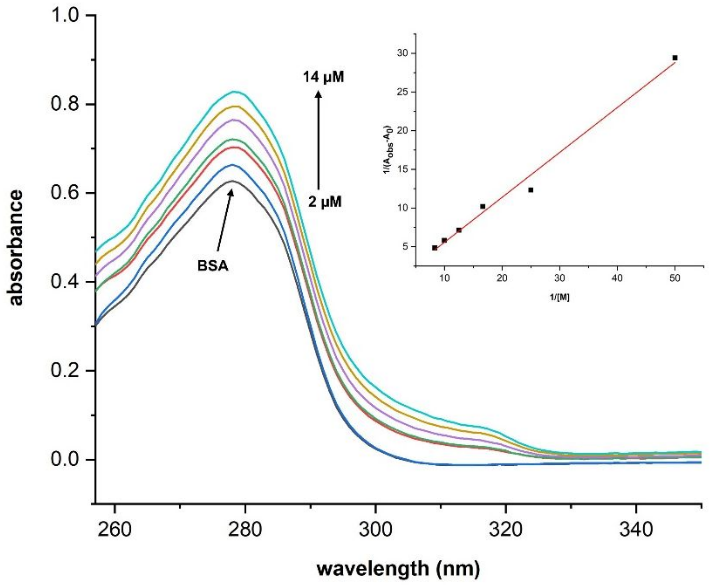


**Figure S17.** The UV-Vis spectra of BSA with increasing concentrations of **2c** solution

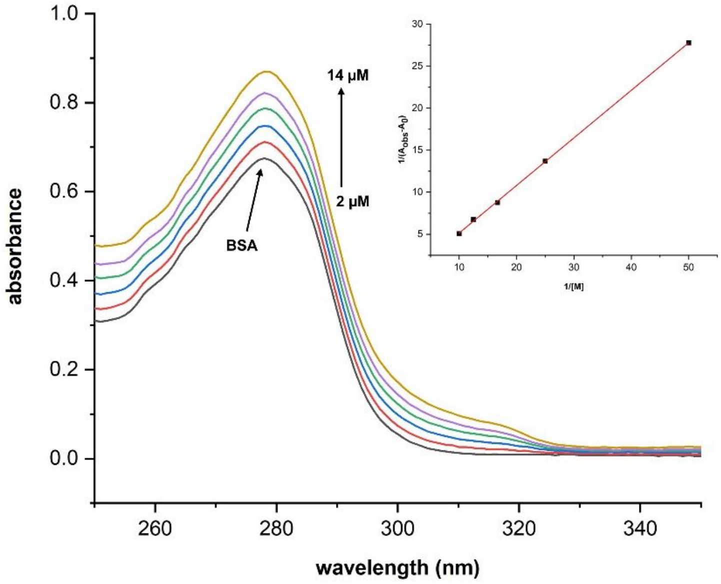


**Figure S18.** The UV-Vis spectra of BSA with increasing concentrations of **2e** solution

Elemental Analysis Results (P1=2a; P2=2b; P3=2c; P4=2d; P5=2e)

**ORDU ÜNİVERSİTESİ**
**MERKEZİ ARAŞTIRMA LABORATUVARI**
Cumhuriyet Mahallesi, Ordu Üniversitesi Cumhuriyet Yerleşkesi 52200, Ordu/TÜRKİYE
Tel: +90 452 226 5200E-Posta:odumaral@odu.edu.trhttp://odumaral.odu.edu.tr/

**ELEMENTEL ANALİZ SONUÇ RAPORU – 28.05.2025**

| Sample Name | Weight [mg] | N % | C % | H % | S % |
|---|---|---|---|---|---|
| P1 | 0.6700 | 8,24 | 43.55 | 4,35 | 0,00 |
| P2 | 0.4950 | 9,90 | 46.68 | 3,23 | 0,00 |
| P3 | 0.6800 | 9,67 | 46.57 | 3,57 | 0,00 |
| P4 | 0.5250 | 8,99 | 47.02 | 4,31 | 0,00 |
| P5 | 0.6740 | 9,10 | 46,55 | 3,92 | 0,00 |

*Elementar Vario Micro Cube*

**Table S1.** Active amino acids residue, binding types and the binding affinity of **2a-e** against HSA and BSA

| Molecules | BA* | Amino Acids Residue |
|---|---|---|
| | | **HSA** |
| **2a** | -4.93 | Cys448 (H-bond), Ala191, Val455 (alkylic interactions), Lys195, Lys436 (pi-interactions), Glu292, Pro447, Asp451, Tyr452 (van der Waals interactions) |
| **2b** | -4.92 | Cys448 (H-bond), Ala191, Val455 (alkylic interactions), Lys195, Lys436 (pi-interactions), Ser192, Glu292, Pro447, Asp451, Tyr452 (van der Waals interactions) |
| **2c** | -5.06 | Cys448 (H-bond), Ala191, Val455 (alkylic interactions), Lys195, Lys436 (pi-interactions), Ser192, Glu292, Pro447, Asp451, Tyr452 (van der Waals interactions) |
| **2d** | -5.18 | Cys448 (H-bond), Ala191, Val455 (alkylic interactions), Lys195, Lys436 (pi-interactions), Glu188, Ser192, Glu292, Pro447, Asp451, Tyr452 (van der Waals interactions) |
| **2e** | -5.90 | Lys195, Glu292, Cys448 (H-bond), Ala191, Val455 (alkylic interactions), Lys436 (pi-interactions), Glu188, Ser192, Pro447, Asp451, Tyr452 (van der Waals interactions) |
| | | **BSA** |
| **2a** | -8.22 | His145, Leu189, Glu424, Ser428 (H-bonds), Arg196, Leu454, Ile455 (alkylic interactions), Ala193, Arg458 (pi-interactions), Thr190, Ser192, Val425, Asn457 (van der Waals interactions) |
| **2b** | -8.16 | His145, Leu189, Ala193, Glu424, Ser428 (H-bonds), Arg196, Leu454, Ile455, Val461 (alkylic interactions), Arg458 (pi-interaction), Thr190, Ser192, Asn457 (van der Waals interactions) |
| **2c** | -7.35 | Ser192, Ser428 (H-bonds), His145, Leu189, Arg196, Val425, Ile455 (alkylic interactions), Ala193, Arg458 (pi-interactions), Asp108, Arg144, Thr190, Glu424, Leu454 (van der Waals interactions) |
| **2d** | -8.48 | Ser428 (H-bond), His145, Pro146, Leu189, Arg196, Val425, Ile455 (alkylic interactions), Ala193, Arg458 (pi-interactions), Asp108, Arg144, Thr190, Ser192, Glu424, Leu454, Asn457 (van der Waals interactions) |
| **2e** | -8.14 | His145, Leu189, Ala193, Glu424, Ser428, Leu454, Arg458 (H-bonds), Arg196, Ile455 (alkylic interactions), Asp108, Thr190, Ser192, Asn457, Val461 (van der Waals interactions) |
